# Cardiovascular–Kidney–Metabolic Syndrome Severity and Risk of Pancreatic Cancer in a Large Prospective Cohort

**DOI:** 10.1002/dmrr.70214

**Published:** 2026-08-04

**Authors:** Munseok Choi, Seok‐Jae Heo, Yu‐Jin Kwon, Chang Moo Kang

**Affiliations:** ^1^ Division of HBP Surgery, Department of Surgery Yonsei University College of Medicine Yongin Severance Hospital Yongin Republic of Korea; ^2^ Department of Biomedical Systems Informatics Yonsei University College of Medicine Seoul Republic of Korea; ^3^ Department of Family Medicine Yonsei University College of Medicine Severance Hospital Seoul Republic of Korea; ^4^ Division of HBP Surgery, Department of Surgery Yonsei University College of Medicine Severance Hospital Seoul Republic of Korea

**Keywords:** cardiovascular–kidney–metabolic syndrome, metabolic dysfunction, pancreatic cancer

## Abstract

**Aims:**

Pancreatic cancer remains one of the most lethal malignancies due to late diagnosis and limited opportunities for prevention. Cardiovascular–kidney–metabolic (CKM) syndrome reflects the cumulative burden of metabolic, renal, and cardiovascular dysfunction, but its association with pancreatic cancer risk has not been well established. We investigated this relationship in a large prospective cohort from the UK Biobank.

**Materials and Methods:**

Participants without pancreatic cancer at baseline (2006–2010) were followed through national cancer and mortality registries until 31 December 2022. CKM syndrome was categorised into five stages (0–4) according to American Heart Association criteria. Incident pancreatic cancer was identified using ICD‐9 and ICD‐10 codes. Fine–Gray subdistribution hazard models accounting for death were used to estimate subdistribution hazard ratios (SHRs) and 95% confidence intervals (CIs), with prespecified subgroup analyses performed.

**Results:**

Among 326,148 participants, 1234 incident pancreatic cancer cases were identified over a mean follow‐up of 13.5 years (SD 2.0). Incidence rates increased progressively from 9.0 to 48.6 per 100,000 person‐years across CKM stages. Compared with CKM stage 0, multivariable‐adjusted SHRs for pancreatic cancer were 1.97 (95% CI 1.28–3.02) for stage 1, 2.02 (1.42–2.87) for stage 2, and 2.08 (1.42–3.04) for stages 3–4 (*p* for trend < 0.001). Associations were generally consistent across subgroup analyses according to age, sex, smoking status, and alcohol consumption, without statistically significant interaction effects.

**Conclusions:**

Advanced CKM syndrome was independently associated with higher pancreatic cancer risk, supporting its potential role in risk stratification and early detection strategies.

**Abbreviations**
AHA: American Heart Association
BMI: Body Mass Index
CKD: Chronic kidney disease
CKM: Cardiovascular–kidney–metabolic
CVD: Cardiovascular disease
eGFR: Estimated glomerular filtration rate
HDL‐C: High‐density lipoprotein cholesterol
IGF: Insulin‐like growth factor
IL‐6: Interleukin‐6
IPMN: Intraductal papillary mucinous neoplasm
KDIGO: Kidney disease: improving global outcomes
LDL‐C: Low‐density lipoprotein cholesterol
MetS: Metabolic syndrome
SHR: subdistribution hazard ratio

## 1 Introduction

Pancreatic cancer is one of the leading causes of cancer‐related mortality worldwide [1]. Despite advances in management, the overall 5‐year survival is approximately 10% [2, 3]. This poor prognosis is mainly driven by nonspecific early symptoms, rapid disease progression, and limited efficacy of current treatments, underscoring the importance of identifying high‐risk individuals for early detection and prevention [4].

Pancreatic carcinogenesis is complex and multifactorial and is influenced by genetic, environmental, and metabolic factors [4]. Among these, chronic metabolic dysfunction and inflammation are recognized as major contributors [5]. Obesity, insulin resistance, and diabetes create a pro‐inflammatory and pro‐tumourigenic environment through adiposity‐driven cytokine production, oxidative stress, endothelial dysfunction, and dysregulated insulin/insulin‐like growth factor (IGF) signalling [5, 6]. These interconnected pathways induce DNA damage, stimulate pancreatic stellate cells, and drive malignant transformation and tumour growth [4].

In parallel with these mechanistic insights, the American Heart Association (AHA) recently proposed the cardiovascular–kidney–metabolic (CKM) syndrome framework, which views metabolic, renal, and cardiovascular disorders as a continuum driven by excess or dysfunctional adiposity [7]. CKM syndrome is marked by insulin resistance, adiposity‐driven inflammation [8], oxidative stress [9], and vascular dysfunction [10], which are pathophysiologic disturbances that considerably overlap with those implicated in pancreatic carcinogenesis. This biological convergence indicates that individuals with a higher CKM burden may be more vulnerable to pancreatic cancer. Although prior studies have demonstrated that greater CKM burden is associated with increased risks of all‐cause and cardiovascular mortality, its association with cancer outcomes, particularly pancreatic cancer, remains unclear [11, 12, 13].

Considering the extensive overlap in the metabolic and inflammatory pathways shared by CKM syndrome and pancreatic carcinogenesis [8, 9], we hypothesised that the CKM stage represents an individual's inherent risk for developing pancreatic cancer. Therefore, we aimed to examine the association between CKM syndrome stage and the occurrence of pancreatic cancer in a large population‐based cohort derived from the United Kingdom (UK) Biobank.

## 2 Methods

### 2.1 Study Population

**FIGURE 1:**
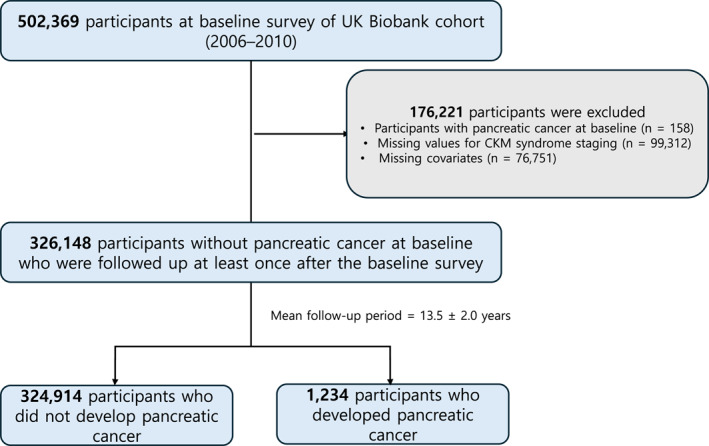
Flow diagram of study participants from the United Kingdom (UK) Biobank cohort.

This study used data from the UK Biobank (https://www.ukbiobank.ac.uk), a prospective cohort of over 500,000 adults aged 40–69 years recruited across the UK between 2006 and 2010. At enrolment, participants completed touchscreen questionnaires, underwent standardized anthropometric and physical evaluations, and provided samples for laboratory analyses. All participants provided written informed consent. The study protocol was approved by the North West Multi‐Centre Research Ethics Committee. Detailed information on UK Biobank design and procedures has been published previously [14]. After excluding individuals with prior pancreatic cancer diagnosis at baseline (*n* = 158), those with incomplete data required for defining CKM syndrome stages (*n* = 99,312), and those with missing covariate data (*n* = 76,751), 326,148 participants without pancreatic cancer and with complete baseline information remained for subsequent analysis. During a mean follow‐up duration of 13.5 ± 2.0 years, 1234 cases of pancreatic cancer were identified, while 324,914 remained cancer‐free. A flow diagram of participant selection is illustrated in Figure 1.

### 2.2 Clinical Variables

Baseline demographic, lifestyle, anthropometric, biochemical, and clinical information was included. Demographic characteristics included age, sex, ethnicity (White or non‐White), Townsend deprivation index, and household income (< £18,000, £18,000–30,999, £31,000–51,999, £52,000–100,000, > £100,000, or unknown). Lifestyle factors included alcohol consumption (never, former, or current), smoking status (never, former, or current), and physical activity quantified as metabolic equivalent (MET) minutes per week. Anthropometric and clinical indices comprised body mass index (BMI, kg/m^2^), waist circumference (WC, cm), systolic and diastolic blood pressure (mmHg), and estimated glomerular filtration rate (eGFR, mL/min/1.73 m^2^) using the Chronic Kidney Disease Epidemiology Collaboration (CKD‐EPI) 2021 equation [15]. Biochemical parameters included HbA1c, fasting glucose, total cholesterol, triglycerides, high‐density lipoprotein cholesterol (HDL‐C), and low‐density lipoprotein cholesterol (LDL‐C). Comorbidities (e.g., hypertension, dyslipidemia, chronic kidney disease [CKD], cardiovascular disease [CVD], cancer, pancreatitis, intraductal papillary mucinous neoplasm [IPMN]) were identified through self‐reports, medication use, or hospital and mortality records using International Classification of Diseases (ICD‐10) codes. Type 1 diabetes mellitus (T1DM) was defined using ICD‐10 code E10. For CKM staging, type 2 diabetes mellitus (T2DM) was defined using ICD‐10 codes E11, E13, or E14, fasting glucose ≥ 126 mg/dL, or HbA1c ≥ 6.5%.

### 2.3 Definition of Cardiovascular–Kidney–Metabolic Syndrome

CKM syndrome was classified into five stages (0–4) following the framework proposed by the AHA, reflecting the increasing involvement of metabolic, renal, and cardiovascular dysfunction [7]. Stage 0 denoted optimal cardiometabolic health, with normal anthropometric measures, glucose and lipid levels, blood pressure, kidney function, and the absence of CVD. Stage 1 included individuals with excess or abnormal adiposity, including overweight, obesity, or central obesity, or prediabetes, but without additional metabolic abnormalities or renal impairment. Stage 2 encompassed participants presenting with at least one metabolic risk factor (e.g., hypertension, diabetes, dyslipidemia, or metabolic syndrome) or moderate‐to‐high‐risk CKD in the absence of CVD.

Stage 3 corresponded to subclinical CVD, defined by very high‐risk CKD (per Kidney Disease: Improving Global Outcomes [KDIGO] criteria) or an estimated 10‐year CVD risk ≥ 20% based on the AHA PREVENT equations [16], together with at least one metabolic or renal abnormality. Stage 4 reflected established CVD, including coronary artery disease, stroke, heart failure, peripheral arterial disease, or atrial fibrillation, identified through hospital or registry records using ICD‐10 codes. Detailed criteria for each CKM stage are provided in Supporting Information S1: Table S1.

### 2.4 Ascertainment of Pancreatic Cancer

Pancreatic cancer cases were identified by linking participants' records to national cancer and mortality registries using ICD‐9 and ICD‐10 codes. ICD‐10 code C25 corresponds to ‘malignant neoplasm of the pancreas’. Additional incident cases were confirmed through participants' self‐reported histories and pancreatic surgery records. The onset date was defined as the earliest record of a pancreatic cancer code within the linked datasets. Participants without evidence of pancreatic malignancy at baseline or during follow‐up were classified as cancer‐free controls. A complete list of diagnostic codes and sources used for identification is provided in Supporting Information S1: Table S2. Although individual‐level histological subtype data were not available through the UK Biobank cancer registry linkage, ICD‐10 code C25 (‘malignant neoplasm of the pancreas’) captures all primary pancreatic malignancies, of which pancreatic ductal adenocarcinoma (PDAC) has been reported to account for approximately 85%–95% in international population‐based registries [17, 18].

### 2.5 Statistical Analysis

Baseline characteristics were expressed as mean ± standard deviation for continuous variables and as frequencies with percentages for categorical variables. Group differences by pancreatic cancer status were assessed using Student's *t*‐tests or chi‐square tests, and differences across CKM syndrome stages were evaluated using one‐way analysis of variance (ANOVA) or chi‐square tests. For the time‐to‐event analysis, the primary outcome was defined as the first diagnosis of pancreatic cancer. The follow‐up period for each participant was calculated from the baseline assessment (2006–2010) to the date of pancreatic cancer diagnosis, death from any cause, or the study end date (31 December 2022), whichever occurred first. Person‐years of follow‐up were then determined based on this duration. The cumulative incidence of pancreatic cancer according to CKM stage at baseline was calculated using the cumulative incidence function, treating death as a competing event. Differences across the CKM stages were tested using Gray's test. The association between CKM syndrome stage and pancreatic cancer risk was assessed using Fine–Gray subdistribution hazard models, treating death from other causes as a competing event. CKM stages 3 and 4 were combined for the primary analyses because stage 3 represented a small proportion of the cohort and included a limited number of pancreatic cancer events, which could result in unstable risk estimates. In addition, both stages represent advanced CKM syndrome characterised by high cardiovascular risk or established CVD, consistent with previous CKM studies [19, 20]. Four models were developed: Model 1 (unadjusted), Model 2 (adjusted for age and sex), Model 3 (further adjusted for smoking status), and Model 4 (additionally adjusted for history of pancreatitis, IPMN, Townsend Deprivation Index, and alcohol consumption). These adjustments accounted for clinically established pancreatic cancer risk factors. A *p* for trend was estimated by modelling CKM stage as a continuous variable. Subgroup analyses were conducted by age (< 50 vs. ≥ 50 years), sex, smoking, and alcohol consumption, with *p* for interaction values calculated. The 50‐year cutoff for age stratification corresponds to the widely accepted definition of early‐onset pancreatic cancer used in recent clinical and epidemiological studies [21, 22]. Sensitivity analyses that excluded participants with any cancer at baseline confirmed the robustness of the results. Additional sensitivity analyses were performed to evaluate the potential influence of reverse causality related to diabetes mellitus. These analyses included exclusion of pancreatic cancer cases occurring within 2 years after diabetes onset and subgroup analyses according to baseline T2DM status. Statistical significance was defined as two‐sided *p*‐values < 0.05. All statistical analyses were conducted using R software (version 4.5.1; R Foundation for Statistical Computing, Vienna, Austria).

## 3 Results

### 3.1 Baseline Characteristics

Table 1 shows baseline characteristics of participants according to pancreatic cancer status. Among 326,148 participants, 1234 participants developed pancreatic cancer over a mean follow‐up of 13.5 years. Participants who developed pancreatic cancer were older and more likely to be men than those who did not (61.1 ± 6.2 vs. 56.3 ± 8.1 years; 55.9% vs. 48.2% men, respectively, both *p* < 0.001) and had a higher proportion of lower household income levels and current smokers (*p* < 0.001 for both). No significant differences in alcohol consumption or physical activity were observed between the groups. The pancreatic cancer group had significantly higher BMI (28.3 ± 5.1 vs. 27.3 ± 4.7 kg/m^2^, *p* < 0.001), WC (94.1 ± 14.1 vs. 90.3 ± 13.4 cm, *p* < 0.001), and metabolic syndrome (MetS) prevalence (26.1% vs. 18.5%, *p* < 0.001) than the control group. Comorbid conditions including diabetes (10.5% vs. 4.9%), hypertension (37.7% vs. 26.2%), dyslipidemia (21.6% vs. 14.6%), CVD (12.1% vs. 8.5%), and malignancy (12.8% vs. 8.9%) were more common in the pancreatic cancer group than in the control group (all *p* < 0.001). Similarly, a history of pancreatitis was more frequently observed in the pancreatic cancer group than in the control group (0.5% vs. 0.1%, *p* < 0.001), whereas IPMN prevalence did not differ significantly between the groups (0.1% vs. 0.0%, *p* = 0.300). Supporting Information S1: Table S3 summarises baseline characteristics by CKM syndrome stage. Baseline characteristics comparing participants who developed pancreatic cancer with those who did not within each CKM syndrome stage are presented in Supporting Information S1: Table S4.

**TABLE 1:** Baseline characteristics of study participants according to the development of pancreatic cancer.

Characteristics	Total	Did not develop pancreatic cancer	Developed pancreatic cancer	*p*‐value	SMD
*n*	326,148	324,914	1234		
Age, year	56.3 ± 8.1	56.3 ± 8.1	61.1 ± 6.2	< 0.001	0.661
Sex, *n* (%)				< 0.001	0.154
Female	168,740 (51.7%)	168,196 (51.8%)	544 (44.1%)		
Male	157,408 (48.3%)	156,718 (48.2%)	690 (55.9%)		
Ethnicity, *n* (%)				0.102	0.051
Non‐white	16,101 (4.9%)	16,053 (4.9%)	48 (3.9%)		
White	310,047 (95.1%)	308,861 (95.1%)	1186 (96.1%)		
Town deprivation index	−1.42 ± 3.02	−1.42 ± 3.02	−1.35 ± 3.17	0.463	0.021
Household income, GBP/year				< 0.001	0.267
< 18,000	60,785 (18.6%)	60,481 (18.6%)	304 (24.6%)		
18,000–30,999	71,834 (22.0%)	71,523 (22.0%)	311 (25.2%)		
31,000–51,999	76,829 (23.6%)	76,552 (23.6%)	277 (22.4%)		
52,000–100,000	62,455 (19.1%)	62,316 (19.2%)	139 (11.3%)		
> 100,000	17,115 (5.2%)	17,070 (5.3%)	45 (3.6%)		
Unknown	37,130 (11.4%)	36,972 (11.4%)	158 (12.8%)		
Alcohol, *n* (%)				0.382	0.038
Never	12,819 (3.9%)	12,769 (3.9%)	50 (4.1%)		
Former	11,185 (3.4%)	11,134 (3.4%)	51 (4.1%)		
Current	302,144 (92.6%)	301,011 (92.6%)	1133 (91.8%)		
Smoking, *n* (%)				< 0.001	0.157
Never	178,085 (54.6%)	177,492 (54.6%)	593 (48.1%)		
Former	114,568 (35.1%)	114,106 (35.1%)	462 (37.4%)		
Current	33,495 (10.3%)	33,316 (10.3%)	179 (14.5%)		
Physical activity, MET‐min/wk	2655.7 ± 2715.5	2655.4 ± 2715.0	2728.0 ± 2830.6	0.369	0.026
BMI, kg/m^2^	27.3 ± 4.7	27.3 ± 4.7	28.3 ± 5.1	< 0.001	0.201
Waist, cm	90.3 ± 13.4	90.3 ± 13.4	94.1 ± 14.1	< 0.001	0.289
SBP, mmHg	137.6 ± 18.5	137.6 ± 18.5	142.2 ± 19.4	< 0.001	0.241
DBP, mmHg	82.3 ± 10.1	82.3 ± 10.1	83.2 ± 10.5	0.002	0.089
HbA1c, %	5.4 ± 0.6	5.4 ± 0.6	5.7 ± 0.8	< 0.001	0.310
Glucose, mg/dl	92.1 ± 21.8	92.1 ± 21.8	97.4 ± 30.1	< 0.001	0.205
TC, mg/dl	220.0 ± 43.9	220.0 ± 43.9	217.2 ± 47.0	0.037	0.061
Triglyceride, mg/dl	154.5 ± 91.0	154.4 ± 91.0	168.2 ± 95.2	< 0.001	0.148
HDL‐C, mg/dl	56.0 ± 14.8	56.0 ± 14.8	54.2 ± 14.8	< 0.001	0.120
LDL‐C, mg/dl	137.5 ± 33.4	137.6 ± 33.4	135.7 ± 35.2	0.068	0.054
eGFR, mL/min/1.73 m^2^	95.8 ± 15.0	95.9 ± 15.0	90.0 ± 14.8	< 0.001	0.395
MetS, *n* (%)	60,562 (18.6%)	60,240 (18.5%)	322 (26.1%)	< 0.001	0.182
Comorbidities, *n* (%)					
Type 1 diabetes	1551 (0.5%)	1541 (0.5%)	10 (0.8%)	0.132	0.042
Type 2 diabetes	15,927 (4.9%)	15,797 (4.9%)	130 (10.5%)	< 0.001	0.214
Hypertension	85,591 (26.2%)	85,126 (26.2%)	465 (37.7%)	< 0.001	0.248
Dyslipidemia	47,757 (14.6%)	47,490 (14.6%)	267 (21.6%)	< 0.001	0.183
CVD	27,679 (8.5%)	27,530 (8.5%)	149 (12.1%)	< 0.001	0.119
Malignancy	29,195 (9.0%)	29,037 (8.9%)	158 (12.8%)	< 0.001	0.124
CKD	815 (0.2%)	808 (0.2%)	7 (0.6%)	0.051	0.050
Heart failure	1630 (0.5%)	1618 (0.5%)	12 (1.0%)	0.031	0.056
PAOD	4356 (1.3%)	4338 (1.3%)	18 (1.5%)	0.800	0.011
Stroke	4956 (1.5%)	4925 (1.5%)	31 (2.5%)	0.006	0.071
Atrial fibrillation	5399 (1.7%)	5366 (1.7%)	33 (2.7%)	0.007	0.070
Hx of pancreatitis	173 (0.1%)	167 (0.1%)	6 (0.5%)	< 0.001	0.084
IPMN	34 (0.0%)	33 (0.0%)	1 (0.1%)	0.300	0.033
CKM syndrome				< 0.001	0.317
Stage 0	26,669 (8.2%)	26,636 (8.2%)	33 (2.7%)		
Stage 1	21,276 (6.5%)	21,219 (6.5%)	57 (4.6%)		
Stage 2	247,278 (75.8%)	246,325 (75.8%)	953 (77.2%)		
Stage 3	4165 (1.3%)	4122 (1.3%)	43 (3.5%)		
Stage 4	26,760 (8.2%)	26,612 (8.2%)	148 (12.0%)		

*Note:* Values are presented as mean ± standard deviation (SD) for continuous variables and number (percentage) for categorical variables. *p*‐values were calculated using the independent two‐sample *t*‐test for continuous variables and the chi‐square test for categorical variables. Comorbidities were identified using hospital admission and registry records in the UK Biobank, based on the following ICD‐10 and OPCS‐4 codes: Hypertension (I10–I13, I15); Dyslipidemia (E78); Cardiovascular disease (I20–I25, I40–I43, I50–I52, I60–I69, I70–I79); Cancer (C00–C97); Chronic kidney disease (CKD: I12–I13, N18, E102, E112, T861, Z940; OPCS‐4 procedure codes: L741–L749, M012–M019, M023, M084, M172, M174, M178, M179, X402, X405, X406, X411, X412); Peripheral arterial occlusive disease (PAOD: I70–I74); Stroke (I60–I64); Atrial fibrillation (I48); History of pancreatitis (K86.0, K86.1), intraductal papillary mucinous neoplasm (D13.6, D37.7). Type 2 diabetes mellitus (T2DM) was defined using ICD‐10 codes E11, E13, or E14 (UK Biobank Fields 130708, 130712, and 130714), fasting glucose ≥ 126 mg/dL (Field 30740), or HbA1c ≥ 6.5% (Field 30750). Type 1 diabetes mellitus (T1DM) was separately defined using ICD‐10 code E10 and was descriptively reported but not included in the operational CKM diabetes definition.

Abbreviations: BMI, body mass index; CKD, chronic kidney disease; CVD, cardiovascular disease; DBP, diastolic blood pressure; eGFR, estimated glomerular filtration rate; HbA1c, glycated haemoglobin; HDL‐C, high‐density lipoprotein cholesterol; IPMN, intraductal papillary mucinous neoplasm; LDL‐C, low‐density lipoprotein cholesterol; MetS, metabolic syndrome; PAOD, peripheral arterial occlusive disease; SBP, systolic blood pressure; SMD, standardized mean differences; TC, total cholesterol.

### 3.2 Cumulative Incidence by CKM Syndrome Stage

**FIGURE 2:**
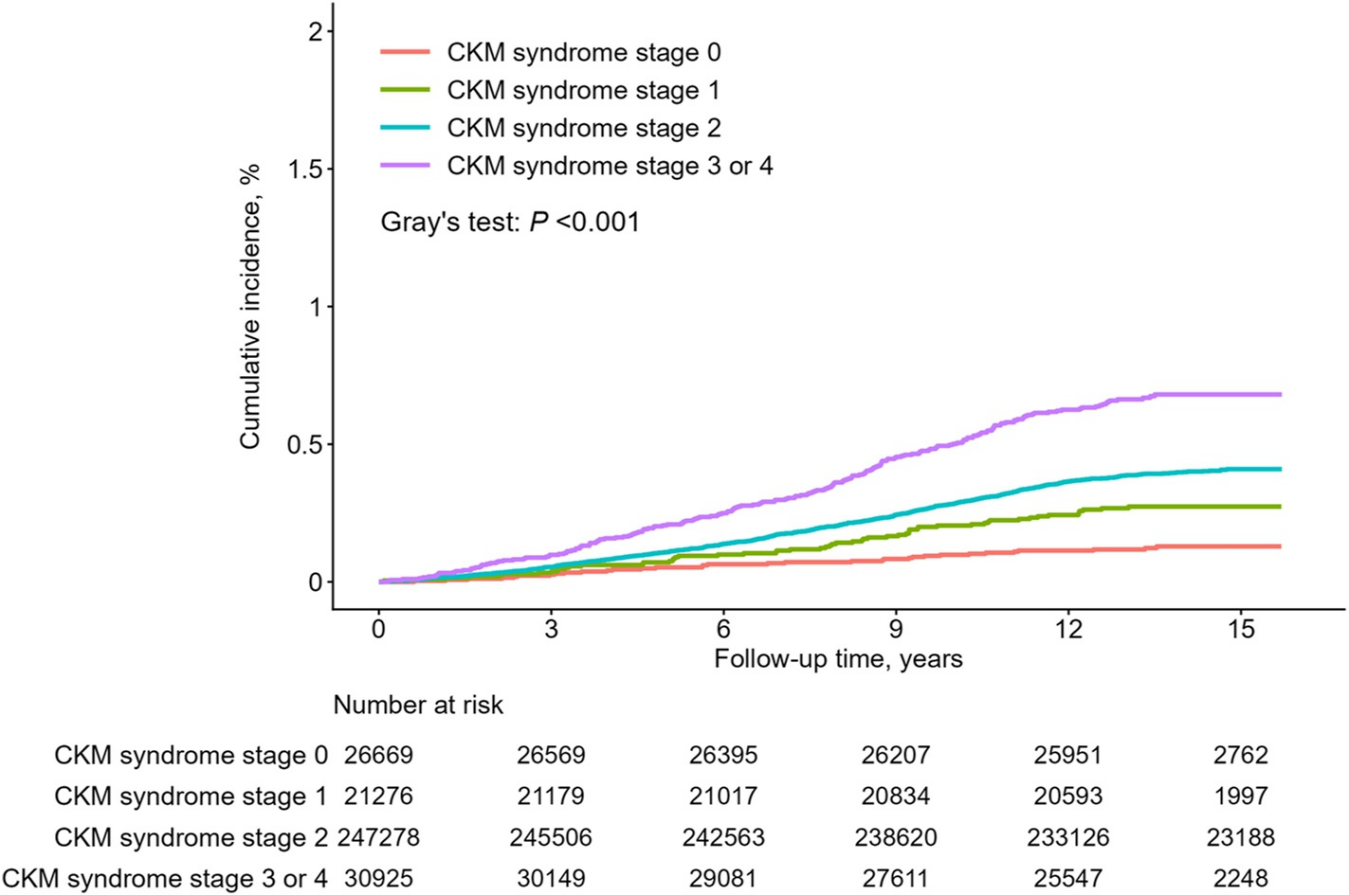
Cumulative incidence of pancreatic cancer by cardiovascular–kidney–metabolic (CKM) syndrome stage. Cumulative incidence of pancreatic cancer according to CKM syndrome stage at baseline was estimated using the cumulative incidence function with death treated as a competing event. Participants with a higher CKM syndrome stage exhibited a significantly greater cumulative incidence of pancreatic cancer than did those with stage 0. Differences across the CKM stages were tested using Gray's test.

Figure 2 illustrates the cumulative incidence of pancreatic cancer by CKM syndrome stage at baseline. Individuals with higher CKM stages showed a progressively greater risk of pancreatic cancer than did those in stage 0, with statistically significant differences across stages (Gray's test *p* < 0.001).

### 3.3 Risk of Pancreatic Cancer According to CKM Syndrome Stage

Table 2 displays the incidence rates and subdistribution hazard ratios (SHRs) for pancreatic cancer across CKM stages. Incidence rates increased with advancing stage, from 9.0 per 100,000 person‐years in stage 0–48.6 per 100,000 person‐years in stages 3–4. In the unadjusted model (Model 1), the risks of pancreatic cancer among participants in CKM stages 1, 2, and 3–4 were 2.17‐fold (95% confidence intervals [CI], 1.41–3.33), 3.12‐fold (95% CI, 2.20–4.41), and 5.00‐fold (95% CI, 3.46–7.24) higher, respectively, than those in stage 0. After adjustment for age, sex, smoking status, history of pancreatitis, IPMN, Townsend Deprivation Index, and alcohol consumption (Model 4), SHRs remained significant at 1.97 (95% CI, 1.28–3.02) for stage 1, 2.02 (95% CI, 1.42–2.87) for stage 2, and 2.08 (95% CI, 1.42–3.04) for stages 3–4. A significant linear trend (*p* for trend < 0.001) persisted across CKM stages, indicating a graded increase in pancreatic cancer risk with higher CKM burden, even after full adjustment.

**TABLE 2:** Incidence rates and multivariable‐adjusted subdistribution hazard ratios of pancreatic cancer according to cardiovascular‐kidney‐metabolic (CKM) syndrome stage at baseline (competing risk: death).

CKM stage	*N*	Events, *n*	Person‐years (PY)	IR^a^	Model 1	*P*	Model 2	*P*	Model 3	*P*	Model 4	*P*
					SHR (95% CI)		SHR (95% CI)		SHR (95% CI)		SHR (95% CI)	
0	26,669	33	366,206	9.011	Reference		Reference		Reference		Reference	
1	21,276	57	290,931	19.592	2.17 (1.41–3.33)	< 0.001	1.99 (1.29–3.05)	0.002	1.97 (1.28–3.03)	0.002	1.97 (1.28–3.02)	0.002
2	247,278	953	3,347,482	28.469	3.12 (2.20–4.41)	< 0.001	2.03 (1.43–2.89)	< 0.001	2.02 (1.42–2.87)	< 0.001	2.02 (1.42–2.87)	< 0.001
3–4	30,925	191	393,072	48.592	5.00 (3.46–7.24)	< 0.001	2.22 (1.51–3.25)	< 0.001	2.13 (1.45–3.11)	< 0.001	2.08 (1.42–3.04)	< 0.001
*p* for trend						< 0.001		< 0.001		< 0.001		< 0.001

*Note:* Model 1: unadjusted. Model 2: adjusted for age and sex. Model 3: adjusted for age, sex, and smoking. Model 4: adjusted for age, sex, smoking, history of chronic pancreatitis, IPMN, Townsend Deprivation Index, and alcohol consumption. *p for trend* was calculated by treating CKM syndrome stage as a continuous variable.

^a^ IR: Pancreatic cancer incidence rate per 100,000 person‐years (PY). Values are presented as number of participants, number of incident pancreatic cancer cases, person‐years of follow‐up, and incidence rate per 100,000 person‐years (PY). Subdistribution hazard ratios (SHRs) and 95% confidence intervals (CIs) were estimated using Fine–Gray subdistribution hazard models with death as a competing event.

When CKM stages were analysed separately, CKM stage 4 also remained significantly associated with pancreatic cancer risk in the fully adjusted model (SHR 1.96, 95% CI 1.33–2.89, *p* < 0.001), although the magnitude of association was lower than that observed for CKM stage 3 (SHR 2.76, 95% CI 1.73–4.42, *p* < 0.001). Nevertheless, the *p* for trend remained statistically significant across all models (*p* for trend < 0.001). Sensitivity analysis yielded results consistent with the primary findings, confirming the reliability of the observed associations. Supporting Information S1: Table S5 summarises the baseline characteristics of participants by pancreatic cancer status, whereas Supporting Information S1: Table S6 summarises the baseline characteristics by CKM stage after excluding individuals with pre‐existing cancer. Supporting Information S1: Table S7 summarises the incidence rates and multivariable‐adjusted SHRs for pancreatic cancer across CKM stages, demonstrating similar patterns.

### 3.4 Risk Factor–Stratified Association Between CKM Stage and Pancreatic Cancer

**FIGURE 3:**
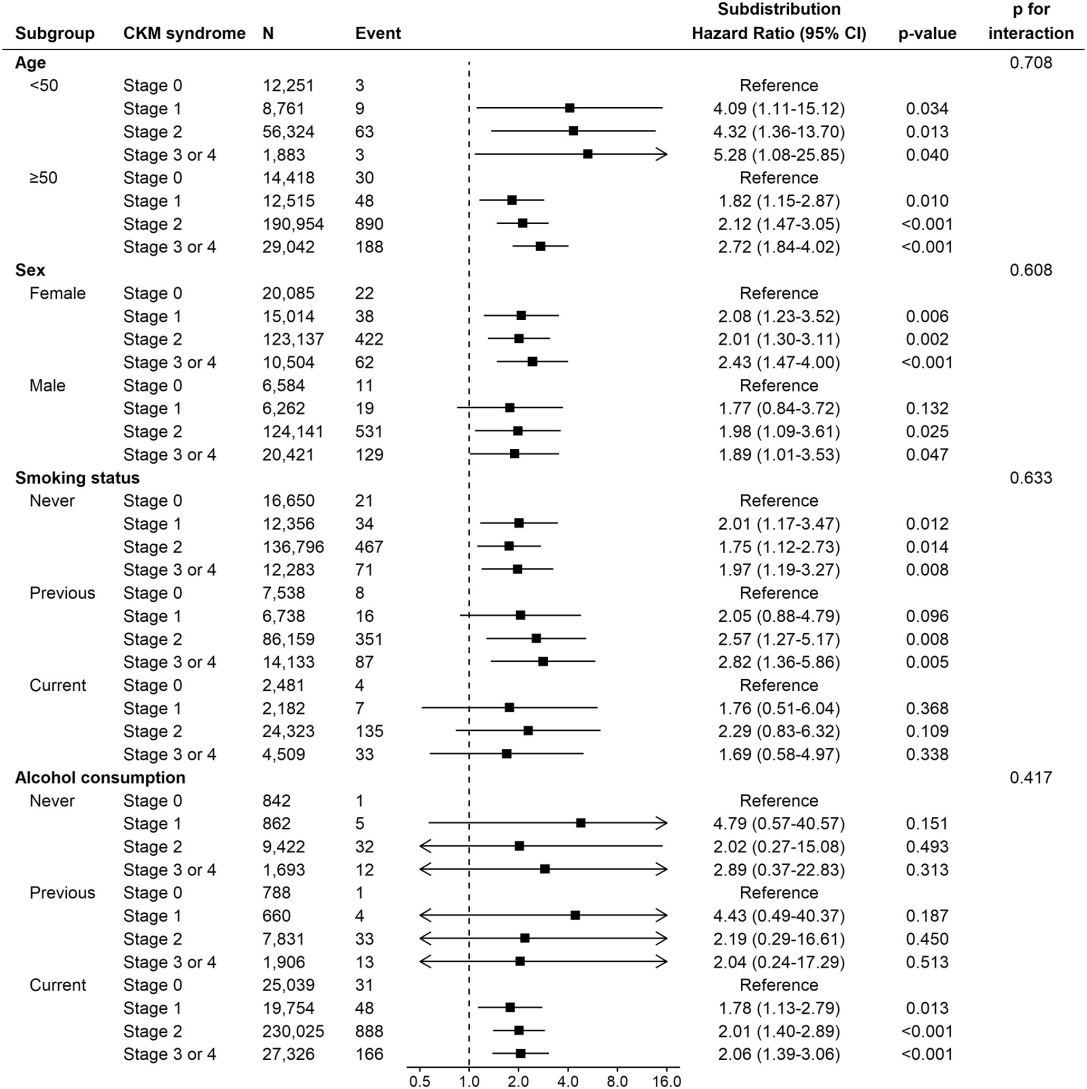
Subgroup analyses of the association between cardiovascular–kidney–metabolic syndrome stage and pancreatic cancer risk subdistribution hazard ratios (SHRs) and 95% confidence intervals (CIs) for pancreatic cancer risk across CKM syndrome stages are presented for predefined subgroups: age (< 50 or ≥ 50 years), sex (women or men), smoking status (never, former, or current), and alcohol consumption (never, former, or current). Analyses were conducted using Fine–Gray subdistribution hazard models, treating death as a competing event, adjusted for age, sex, smoking, history of pancreatitis, intraductal papillary mucinous neoplasm (IPMN), Townsend Deprivation Index, and alcohol consumption except for the variable used as the stratification factor in each respective model. Interaction *p* values were computed to assess whether associations differed significantly across subgroups.

Figure 3 illustrates the subgroup analyses of the association between CKM syndrome stage and pancreatic cancer risk. No significant interactions were observed between CKM stage and age, sex, smoking status, or alcohol consumption (all *p* for interaction > 0.05), suggesting generally consistent associations across subgroups. The association was evident in both age groups but was stronger in participants aged < 50 years (SHR 5.28; 95% CI, 1.08–25.85) than in those aged ≥ 50 years (SHR 2.72; 95% CI, 1.84–4.02); the wide confidence interval in the younger group reflects the small number of events in this subgroup. By sex, significant associations between advanced CKM stage (stage 3–4) and pancreatic cancer risk were observed in both women and men. In the fully adjusted model, the subdistribution hazard ratio was 2.43 (95% CI, 1.47–4.00; *p* < 0.001) in women and 1.89 (95% CI, 1.01–3.53; *p* = 0.047) in men, although the magnitude of association appeared stronger in women.

For smoking, higher CKM stages were associated with an increased risk of pancreatic cancer among never smokers and former smokers, whereas this association was not significant among current smokers. Among alcohol consumption groups, significant associations were observed in current drinkers (SHR for stage 3–4, 2.06 [95% CI, 1.39–3.06]), whereas the associations in non‐drinkers or former drinkers were not significant.

### 3.5 Survival Outcomes According to Surgical Operability

Survival outcomes were further compared according to surgical operability among participants with pancreatic cancer (Supporting Information S1: Table S8). Individuals who underwent surgery (‘Op’) showed substantially lower hazards of death than did those who did not undergo surgery (‘Non‐op’) across all CKM stages. In the overall cohort (CKM stages 1–4), the adjusted hazard ratio for death in the Op group relative to the Non‐op group was 0.38 (95% CI, 0.33–0.45), and a similar pattern was observed across individual CKM stages: stage 1 (HR 0.27, 95% CI, 0.13–0.53), stage 2 (HR 0.38, 95% CI, 0.32–0.46), and stages 3–4 (HR 0.34, 95% CI, 0.22–0.55), demonstrating the survival benefit associated with surgical resection across CKM stages.

### 3.6 Additional Analyses Related to Diabetes Mellitus

Given the established relationship between diabetes mellitus and pancreatic cancer, additional analyses were performed to evaluate potential reverse causality and the influence of baseline T2DM. Among pancreatic cancer cases with diabetes, the mean duration of diabetes prior to pancreatic cancer diagnosis was 8.49 years (SD 9.46), with a median duration of 5.87 years (interquartile range 0.35–13.17 years). Exclusion of pancreatic cancer cases occurring within 2 years after diabetes onset did not materially alter the observed associations between CKM stage and pancreatic cancer risk (Supporting Information S1: Table S9). Additional subgroup analyses according to baseline T2DM status demonstrated persistent associations between CKM stage and pancreatic cancer risk among participants without baseline diabetes mellitus, whereas progression from CKM stage 2 to stages 3–4 was not associated with a significant additional increase in pancreatic cancer risk among participants with baseline T2DM (Supporting Information S1: Table S10).

## 4 Discussion

This study showed a dose–response relationship between CKM syndrome stage and pancreatic cancer incidence. The risk increased progressively across CKM stages, with the highest risk being in stages 3–4, where metabolic, renal, and cardiovascular dysfunctions co‐exist.

MetS is significantly associated with pancreatic cancer risk [23, 24, 25]. In a nationwide cohort study from Korea, Park et al. reported that MetS and its dynamic changes were strongly associated with pancreatic cancer risk. Individuals with persistent MetS exhibited the highest risk, followed by those who developed MetS and those who recovered from it, compared with those who remained MetS‐free [24]. Recovery from MetS was associated with a reduced, but not fully normalised, pancreatic cancer risk, suggesting that the elevated risk associated with prior metabolic dysfunction may persist even after metabolic improvement [24]. Similarly, in a large Japanese cohort, Miyashita et al. found that MetS and even pre‐MetS, defined by central obesity plus one additional metabolic abnormality, were associated with higher pancreatic cancer incidence [23]. The risk rose stepwise with the number of MetS components, implying that early metabolic abnormalities may initiate tumour‐promoting pathways before overt diabetes or CVD develops [23]. A meta‐analysis by Zhong et al. further supported these findings, reporting a pooled relative risk of 1.34 (95% CI, 1.23–1.46) for pancreatic cancer among individuals with MetS, with hyperglycemia, hypertension, and low HDL cholesterol identified as key contributors [25].

Although CKD and CVD have been linked to higher overall cancer incidence and mortality in prior cohort studies, evidence specific to pancreatic cancer has been limited. One nationwide study reported a stepwise rise in pancreatic cancer risk with declining eGFR, implicating systemic inflammation, oxidative stress, uraemic toxin accumulation, and impaired immune surveillance as potential mechanisms [26]. Similarly, individuals with pre‐existing CVDs, including coronary artery disease and heart failure (HF), show increased risks of malignancy, likely mediated by chronic inflammation, vascular injury, and metabolic stress [10]. Two population‐based studies have documented increased overall cancer risk among individuals with CVD or HF; however, pancreatic cancer was assessed only as part of secondary, site‐specific subgroup analyses rather than as a primary outcome [27, 28]. Therefore, despite epidemiologic links between metabolic disorders and pancreatic cancer, direct evidence linking CKD or CVD to pancreatic carcinogenesis remains limited.

Our study applied an integrated CKM framework that encompasses metabolic, renal, and cardiovascular dysfunction. These domains had limited prior evidence linking them to pancreatic cancer. A clear dose–response association was identified between CKM stage and pancreatic cancer risk, with the highest risk observed at stages 3–4, where multiorgan metabolic, inflammatory, and vascular disturbances emerge (Supporting Information S1: Figure S1).

Several biological mechanisms may underlie the association between CKM syndrome and pancreatic cancer. In the early CKM stages, insulin resistance and compensatory hyperinsulinemia may induce pancreatic carcinogenesis by overstimulating insulin and IGF signalling, driving acinar and ductal cell proliferation, enhanced angiogenesis, reduced apoptosis, and increased local inflammation [29]. Visceral adiposity and ectopic lipid buildup in the pancreas and liver further increase metabolic stress and lipotoxicity, causing chronic low‐grade inflammation mediated by tumour necrosis factor‐α (TNF‐α), interleukin‐6 (IL‐6), and leptin, along with reduced anti‐inflammatory adiponectin [8, 9]. These cytokine‐driven inflammatory pathways elevate oxidative stress and reactive oxygen species, damage DNA, impair tumour‐suppressor pathways, such as p53, and create a pro‐inflammatory microenvironment that supports KRAS‐mutant cell survival and signalling, key steps in pancreatic carcinogenesis [30, 31, 32]. As CKM advances, kidney dysfunction and vascular injury contribute to systemic inflammation, uraemic toxin buildup, and endothelial dysfunction [26, 27, 28]. These processes intensify oxidative stress and impair immune surveillance, reinforcing a pro‐tumourigenic milieu that can further promote both the development and progression of pancreatic cancer [26, 27, 28].

CKM syndrome was associated with a higher incidence of pancreatic cancer. Among patients who developed pancreatic cancer, those who underwent surgical resection showed substantially better survival outcomes across all CKM stages. These findings suggest that operability was associated with improved survival across CKM stages. However, because formal comparisons of prognosis across CKM stages were not specifically performed, further studies are needed to clarify whether CKM syndrome independently influences pancreatic cancer survival outcomes.

Subgroup analyses generally reinforced the consistency of the main findings. The association was observed in both age groups, with greater precision among participants aged ≥ 50 years due to a larger number of events. Although no statistically significant interactions were observed according to age, sex, smoking status, or alcohol consumption, some numerical differences in hazard ratios were noted across strata. By sex, the association appeared more pronounced in women than in men, which may relate to postmenopausal hormonal changes and inflammatory susceptibility [33].

The attenuated association observed among current smokers may reflect the dominant carcinogenic effect of active smoking, which could partially overshadow CKM‐related metabolic risk [34]. Conversely, the relatively preserved association among never and former smokers raises the possibility that CKM staging may provide additional clinical value for identifying elevated pancreatic cancer risk even among individuals traditionally considered at lower risk. Similarly, the numerically stronger association observed among current drinkers may suggest a potential interaction between alcohol‐related oxidative stress, pancreatic inflammation, and metabolic dysregulation [35, 36]. Although these subgroup findings should be interpreted cautiously and considered exploratory, they support the consistency of the association between CKM syndrome and pancreatic cancer risk across demographic and lifestyle strata.

An additional finding of interest was the numerically lower risk estimate observed for CKM stage 4 compared with stage 3 when stages were analysed separately. Although CKM stage 4 remained significantly associated with pancreatic cancer risk, the magnitude of association was numerically lower than that observed for stage 3. Because confidence intervals overlapped substantially and formal comparisons between advanced CKM stages were not specifically performed, this numerical difference should be interpreted cautiously. Nevertheless, the heterogeneous clinical composition of CKM stage 4, including advanced cardiovascular and renal disease, may partially contribute to differences in observed risk estimates across advanced CKM stages.

Because diabetes mellitus is both a component of CKM syndrome and an established risk factor for pancreatic cancer, we also performed additional analyses to further evaluate its potential contribution to the observed associations. Importantly, the exclusion of pancreatic cancer cases occurring shortly after diabetes onset did not alter the results, reducing concerns regarding substantial reverse causality from occult pancreatic cancer–associated diabetes. Furthermore, elevated pancreatic cancer risk remained among participants without baseline diabetes, although the risk gradient across higher CKM stages was attenuated after multivariable adjustment. These findings suggest that the association between CKM syndrome and pancreatic cancer risk is not solely explained by overt diabetes alone, although diabetes itself may play an important role within the CKM continuum. This study has some limitations. First, selection bias is possible because UK Biobank participants tend to be healthier than the general population, potentially underestimating the true risk of pancreatic cancer. Second, the CKM stage was assessed only at baseline and was not updated during follow‐up. Therefore, longitudinal changes in metabolic, renal, and cardiovascular status could not be captured, and some participants may have progressed to more advanced CKM stages over time, potentially resulting in exposure misclassification and underestimation of the true association between advanced CKM syndrome and pancreatic cancer risk. In addition, because of the observational nature of the study, reverse causality cannot be completely excluded. Third, individual‐level histological subtype and tumour‐specific staging data were not accessible through the UK Biobank cancer registry linkage. However, because PDAC consistently accounts for approximately 85%–95% of all pancreatic malignancies in international population‐based registries [17, 18], the majority of incident pancreatic cancer cases identified by ICD‐10 code C25 in our cohort were likely to represent PDAC. Nonetheless, subtype‐specific analyses were not feasible, and future studies incorporating histopathological data are warranted to validate our findings. Fourth, residual confounding from unmeasured factors such as diet, medication use, family history, and genetic background may remain. However, the large sample size, prospective design, and consistent dose–response relationship enhance the validity of our findings. Fifth, several subgroup analyses, particularly those among participants younger than 50 years, were based on a small number of events (e.g., only three events in both the stage 0 and stage 3 or 4 groups), resulting in wide confidence intervals (e.g., SHR 5.28 [95% CI, 1.08–25.85]). Although the point estimates appeared numerically larger in this subgroup, the test for interaction by age was not statistically significant (*p* for interaction = 0.708), providing no clear evidence of effect modification, though the limited number of events may have reduced the power to detect such an interaction. These estimates should therefore be interpreted with caution and confirmed in larger studies with longer follow‐up.

To our knowledge, this is the first large‐scale population‐based study to show an association between CKM syndrome and pancreatic cancer risk. With the rapid global rise in CKM syndrome prevalence and the increasing burden of pancreatic cancer, these findings highlight the potential clinical importance of integrating cardiometabolic health assessment and management into future pancreatic cancer prevention strategies. Such integration may be achieved through CKM stage–based risk stratification, optimization of obesity and metabolic risk factor management, and identification of individuals who may benefit from closer surveillance. Future developments may include incorporation of CKM staging into pancreatic cancer risk prediction models and evaluation of whether targeted cardiometabolic interventions can reduce pancreatic cancer risk across the CKM continuum.

## 5 Conclusions

Higher CKM stage was associated with greater pancreatic cancer risk and may represent a potentially modifiable risk factor, highlighting the importance of integrated management of cardiovascular, renal, and metabolic health that may be relevant to pancreatic cancer risk stratification; interventional studies will be needed to establish whether this relationship is causal. Notably, the relatively preserved association observed among non‐smokers raises the possibility that CKM‐based risk assessment may help identify individuals at elevated pancreatic cancer risk even among populations traditionally considered at lower risk, although further validation is required.

## Author Contributions

**Munseok Choi:** conceptualization, investigation, methodology, validation, writing – original draft. **Seok‐Jae Heo:** data curation, formal analysis, software, visualization, writing – original draft. **Yu‐Jin Kwon:** conceptualization, investigation, methodology, project administration, validation, writing – original draft. **Chang Moo Kang:** conceptualization, methodology, supervision, writing – review and editing.

## Funding

This study was supported by a new faculty research seed money grant of Yonsei University College of Medicine for 2026 (Grant 2026‐32‐0061).

## Ethics Statement

All participants provided written informed consent. The study protocol was approved by the North West Multi‐Centre Research Ethics Committee, as well as by the Institutional Review Board of Yongin Severance Hospital (IRB No. 9‐2025‐0145), and was conducted in accordance with the principles of the Declaration of Helsinki.

## Consent

All authors have read and agreed to the published version of the manuscript.

## Conflicts of Interest

The authors declare no conflicts of interest.

## Supporting information


Supporting Information S1

## Data Availability

The data used in this study are available from UK Biobank (https://www.ukbiobank.ac.uk) upon application and approval, in accordance with UK Biobank's data access policies.
