# Supplementary material for: Cardiovascular–Kidney–Metabolic Syndrome Severity and Risk of Pancreatic Cancer in a Large Prospective Cohort
Source: Diabetes Metab Res Rev. 2026 Aug 4;42(6):e70214. doi: 10.1002/dmrr.70214 (PMC13435347; doi:10.1002/dmrr.70214)
Supplement: Supplementary file 1 [file DMRR-42-e70214-s001.docx]

**Supplementary materials**

**Cardiovascular–Kidney–Metabolic Syndrome Severity and Risk of Pancreatic Cancer in a Large Prospective Cohort**

**Table S1. Definition of Cardiovascular-kidney-metabolic (CKM) syndrome**

**Table S2. Data fields for Pancreatic cancer**

**Table S3. Baseline characteristics of study participants according to CKM syndrome stage at baseline**

**Table S4. Baseline characteristics according to pancreatic cancer development within each CKM syndrome stage**

**Table S5. Baseline characteristics of study participants according to the development of pancreatic cancer (excluding baseline all cancer cases)**

**Table S6. Baseline characteristics of study participants according to CKM syndrome stage at baseline (excluding baseline all cancer cases)**

**Table S7. Incidence rates and multivariable-adjusted subdistribution hazard ratios of pancreatic cancer by CKM syndrome stage at baseline (competing risk: death; baseline all cancer excluded)**

**Table S8. Association between surgical operability and outcomes in CKM Patients with pancreatic cancer**

**Table S9. Sensitivity analyses of the association between CKM syndrome stage and pancreatic cancer risk after exclusion of pancreatic cancer cases occurring within 2 years after diabetes onset**

**Table S10. Additional subgroup and sensitivity analyses evaluating the influence of baseline diabetes status on the association between CKM syndrome stage and pancreatic cancer risk**

**Supplementary Figure 1. Conceptual Framework Linking CKM Severity and Pancreatic Cancer Development**

**Table S1. Definition of Cardiovascular-kidney-metabolic (CKM) syndrome**

| **CKM Stage^a^** | **Definition** | **Diagnostic Criteria** | **Representative thresholds** |
| --- | --- | --- | --- |
| Stage 0: Optimal cardiometabolic health | Individuals showing normal body composition, blood pressure, glucose and lipid levels, kidney function, and no evidence of cardiovascular disease. | All of the listed criteria are satisfied. | • BMI < 25 kg/m²  • Waist < 88 cm (women) or < 102 cm (men)  • HbA1c < 5.7% with no history or treatment of diabetes  • SBP < 130 mmHg and DBP < 80 mmHg without antihypertensive medication  • Triglycerides < 135 mg/dL  • CKD classified as low risk by KDIGO (based on eGFR and UACR)  • Estimated 10-year CVD risk < 20%  • No clinical CVD evidence |
| Stage 1: Adiposity or prediabetic state | Participants with excess or dysfunctional adiposity (overweight, obesity, or abdominal obesity) or prediabetes, but without other metabolic risk factors or CKD. | Presence of any one among overweight/obesity, abdominal obesity, or prediabetes. | • SBP < 130 mmHg and DBP < 80 mmHg without antihypertensive use  • Triglycerides < 135 mg/dL  • CKD low-risk category by KDIGO  • 10-year CVD risk < 20%  • Absence of clinical CVD |
| Stage 2: Metabolic risk or kidney dysfunction | Individuals with metabolic abnormalities such as hypertension, dyslipidemia, diabetes, or metabolic syndrome, or those with moderate-to-high CKD risk. | Meeting any of the following: hypertension, hypertriglyceridemia, diabetes, metabolic syndrome, or moderate-to-high-risk CKD. | • No very-high-risk CKD  • 10-year CVD risk < 20%  • No established CVD |
| Stage 3: Subclinical cardiovascular involvement | Individuals with metabolic dysfunction or CKD who show evidence of subclinical CVD. | Either very-high-risk CKD (per KDIGO) or an estimated 10-year CVD risk ≥ 20%. | • At least one CKM component (e.g., obesity, prediabetes, hypertension, diabetes, metabolic syndrome, CKD)  • No overt CVD |
| Stage 4: Clinical cardiovascular disease | Individuals with established clinical CVD in the presence of metabolic abnormalities or CKD. | Diagnosis of clinical cardiovascular disease. | • One or more CKM-related components (e.g., obesity, prediabetes, hypertension, diabetes, metabolic syndrome, or CKD of moderate-to-very-high risk) |

Abbreviations: CKM, cardiovascular–kidney–metabolic; BMI, body mass index; SBP, systolic blood pressure; DBP, diastolic blood pressure; CKD, chronic kidney disease; eGFR, estimated glomerular filtration rate; UACR, urinary albumin-creatinine ratio; HDL-C, high-density lipoprotein cholesterol; CVD, cardiovascular disease; ICD-10, International Classification of Diseases, 10th Revision.

^a^ CKM syndrome stages were defined according to the American Heart Association framework, reflecting the sequential involvement of metabolic, renal, and cardiovascular dysfunction.

1. Anthropometric measures: BMI (Field 21001) ≥ 25 kg/m² (≥ 23 kg/m² for Asian ancestry) or waist circumference (Field 48) ≥ 102 cm (men) / ≥ 88 cm (women); ≥ 90 cm (men) / ≥ 80 cm (women) for Asian ancestry.

2. Prediabetes: Fasting glucose 100–125 mg/dL (Field 30740) or HbA1c 5.7–6.4% (Field 30750) without diabetes diagnosis.

3. Diabetes mellitus: ICD-10 E11, E13, E14 (Fields 130708, 130712, 130714), fasting glucose ≥ 126 mg/dL (Field 30740), or HbA1c ≥ 6.5% (Field 30750).

4. Hypertension: SBP ≥ 130 mmHg or DBP ≥ 80 mmHg (Fields 93, 94, 4080, 4079) or diagnosis of essential hypertension (ICD-10 I10-I15; Field 131286, 131288, 131290, 131292, 131294).

5. Dyslipidemia: Triglycerides ≥ 135 mg/dL (Field 30870) or HDL-C < 40 mg/dL (men) / < 50 mg/dL (women) (Field 30760).

6. Metabolic syndrome: ≥ 3 of the following—abdominal obesity, low HDL-C, triglycerides ≥ 150 mg/dL, SBP ≥ 130 mmHg or DBP ≥ 80 mmHg (or on antihypertensive therapy), and fasting glucose ≥ 100 mg/dL.

7. CKD classification (KDIGO 2021): eGFR calculated from serum creatinine (Field 30703) and/or cystatin C (Field 30720); UACR (Field 30500, 30510). Moderate-to-high-risk CKD = eGFR 30–59 mL/min/1.73 m² or UACR 30–300 mg/g; very-high-risk CKD = eGFR < 30 mL/min/1.73 m² or UACR > 300 mg/g.

8. Subclinical CVD: Presence of very-high-risk CKD or predicted 10-year CVD risk ≥ 20%, estimated using the AHA PREVENT equations.

9. Clinical CVD (Stage 4): Defined using hospital and registry data with ICD-10 codes as follows—
 • Coronary heart disease (I20–I25): 131296 (I20, angina pectoris), 131298 (I21, acute myocardial infarction), 131300 (I22, subsequent MI), 131302 (I23, post-MI complications), 131304 (I24, other acute ischaemic heart diseases), 131306 (I25, chronic ischaemic heart disease);
 • Heart failure: 131354 (I50);
 • Stroke (I60–I64): 131360 (I60, subarachnoid haemorrhage), 131362 (I61, intracerebral haemorrhage), 131364 (I62, other non-traumatic intracranial haemorrhage), 131366 (I63, cerebral infarction), 131368 (I64, unspecified stroke);
 • Peripheral arterial disease (I70–I74): 131380 (I70, atherosclerosis), 131382 (I71, aortic aneurysm and dissection), 131384 (I72, other aneurysm), 131386 (I73, other peripheral vascular diseases), 131388 (I74, arterial embolism and thrombosis);
 • Atrial fibrillation and flutter: 131350 (I48).

10. Stage summary: Stage 0 = optimal cardiometabolic health; Stage 1 = adiposity or prediabetes only; Stage 2 = metabolic risk or moderate-to-high CKD; Stage 3 = subclinical CVD (very-high CKD or ≥ 20% CVD risk); Stage 4 = clinical CVD.

11. UK Biobank variable IDs were obtained from the *Data Showcase*(https://biobank.ndph.ox.ac.uk/showcase/).

**Table S2. Data fields for Pancreatic cancer**

| ICD-10 codes | |
| --- | --- |
| Code | Description |
| C25.0 | Head of pancreas |
| C25.1 | Body of pancreas |
| C25.2 | Tail of pancreas |
| C25.3 | Pancreatic duct |
| C25.4 | Endocrine pancreas |
| C25.7 | Other parts of pancreas |
| C25.8 | Overlapping lesion of pancreas |
| C25.9 | Pancreas, unspecified |
| ICD-9 codes | |
| 1570 | Malignant neoplasm of head of pancreas |
| 1571 | Malignant neoplasm of body of pancreas |
| 1572 | Malignant neoplasm of tail of pancreas |
| 1573 | Malignant neoplasm of pancreatic duct |
| 1578 | Malignant neoplasm of pancreas, other |
| 1579 | Malignant neoplasm of pancreas, part unspecified |
| Self-reported |  |
| 1026 | Pancreas cancer |
| Operative procedures | |
| J55.1 | Total pancreatectomy and excision of surrounding tissue |
| J55.2 | Total pancreatectomy NEC |
| J55.8 | Other specified total excision of pancreas |
| J56.1 | Pancreaticoduodenectomy and excision of surrounding tissue |
| J56.2 | Pancreaticoduodenectomy and resection of antrum of stomach |
| J56.3 | Pancreaticoduodenectomy NEC |
| J56.4 | Subtotal excision of head of pancreas with preservation of duodenum and drainage HFQ |
| J56.8 | Other specified excision of head of pancreas |
| J56.9 | Unspecified excision of head of pancreas |
| J57.1 | Subtotal pancreatectomy |
| J57.2 | Left pancreatectomy and drainage of pancreatic duct |
| J57.4 | Excision of tail of pancreas and drainage of pancreatic duct |
| J57.5 | Excision of tail of pancreas NEC |
| J57.8 | Other specified other partial excision of pancreas |
| J57.9 | Unspecified other partial excision of pancreas |

ICD-10 code C25 corresponds to malignant neoplasm of the pancreas.

**Table S3. Baseline characteristics of study participants according to CKM syndrome stage at baseline**

| **Characteristics** | **Total** | **CKM syndrome stage 0** | **CKM syndrome stage 1** | **CKM syndrome stage 2** | **CKM syndrome stage 3 or 4** | ***P*-value** | **SMD** |
| --- | --- | --- | --- | --- | --- | --- | --- |
| n | 326,148 | 26,669 | 21,276 | 247,278 | 30,925 |  |  |
| Age, year | 56.3 ± 8.1 | 51.6 ± 7.7 | 52.5 ± 7.9 | 56.5 ± 7.9 | 61.7 ± 6.3 | <0.001 | 0.782 |
| Sex, n (%) |  |  |  |  |  | <0.001 | 0.519 |
| Female | 168,740 (51.7%) | 20,085 (75.3%) | 15,014 (70.6%) | 123,137 (49.8%) | 10,504 (34.0%) |  |  |
| Male | 157,408 (48.3%) | 6,584 (24.7%) | 6,262 (29.4%) | 124,141 (50.2%) | 20,421 (66.0%) |  |  |
| Ethnicity, n (%) |  |  |  |  |  | <0.001 | 0.072 |
| Non-white | 16,101 (4.9%) | 1,001 (3.8%) | 1,482 (7.0%) | 12,101 (4.9%) | 1,517 (4.9%) |  |  |
| White | 310,047 (95.1%) | 25,668 (96.2%) | 19,794 (93.0%) | 235,177 (95.1%) | 29,408 (95.1%) |  |  |
| Town deprivation index | -1.4 ± 3.0 | -1.5 ± 3.0 | -1.3 ± 3.0 | -1.5 ± 3.0 | -0.9 ± 3.3 | <0.001 | 0.097 |
| Household income, GBP/year |  |  |  |  |  | <0.001 | 0.410 |
| <18,000 | 60,785 (18.6%) | 3,129 (11.7%) | 2,970 (14.0%) | 44,603 (18.0%) | 10,083 (32.6%) |  |  |
| 18,000–30,999 | 71,834 (22.0%) | 4,808 (18.0%) | 4,128 (19.4%) | 55,396 (22.4%) | 7,502 (24.3%) |  |  |
| 31,000–51,999 | 76,829 (23.6%) | 6,669 (25.0%) | 5,502 (25.9%) | 59,493 (24.1%) | 5,165 (16.7%) |  |  |
| 52,000–100,000 | 62,455 (19.1%) | 6,923 (26.0%) | 5,004 (23.5%) | 47,501 (19.2%) | 3,027 (9.8%) |  |  |
| >100,000 | 17,115 (5.2%) | 2,529 (9.5%) | 1,602 (7.5%) | 12,227 (4.9%) | 757 (2.4%) |  |  |
| Unknown | 37,130 (11.4%) | 2,611 (9.8%) | 2,070 (9.7%) | 28,058 (11.3%) | 4,391 (14.2%) |  |  |
| Alcohol, n (%) |  |  |  |  |  | <0.001 | 0.105 |
| Never | 12,819 (3.9%) | 842 (3.2%) | 862 (4.1%) | 9,422 (3.8%) | 1,693 (5.5%) |  |  |
| Former | 11,185 (3.4%) | 788 (3.0%) | 660 (3.1%) | 7,831 (3.2%) | 1,906 (6.2%) |  |  |
| Current | 302,144 (92.6%) | 25,039 (93.9%) | 19,754 (92.8%) | 230,025 (93.0%) | 27,326 (88.4%) |  |  |
| Smoking, n (%) |  |  |  |  |  | <0.001 | 0.244 |
| Never | 178,085 (54.6%) | 16,650 (62.4%) | 12,356 (58.1%) | 136,796 (55.3%) | 12,283 (39.7%) |  |  |
| Former | 114,568 (35.1%) | 7,538 (28.3%) | 6,738 (31.7%) | 86,159 (34.8%) | 14,133 (45.7%) |  |  |
| Current | 33,495 (10.3%) | 2,481 (9.3%) | 2,182 (10.3%) | 24,323 (9.8%) | 4,509 (14.6%) |  |  |
| Physical activity, MET-min/wk | 2,655.7 ± 2,715.5 | 2,725.0 ± 2,580.0 | 2,649.9 ± 2,625.2 | 2,667.0 ± 2,736.9 | 2,510.0 ± 2,713.4 | <0.001 | 0.041 |
| BMI, kg/m² | 27.3 ± 4.7 | 22.4 ± 1.7 | 26.9 ± 3.3 | 27.6 ± 4.6 | 29.1 ± 5.0 | <0.001 | 1.003 |
| Waist, cm | 90.2 ± 13.4 | 75.7 ± 7.5 | 86.1 ± 10.1 | 91.3 ± 12.9 | 97.4 ± 13.7 | <0.001 | 1.073 |
| SBP, mmHg | 137.6 ± 18.5 | 115.8 ± 8.3 | 117.7 ± 7.6 | 141.0 ± 16.9 | 142.5 ± 20.9 | <0.001 | 1.211 |
| DBP, mmHg | 82.3 ± 10.1 | 70.8 ± 5.6 | 72.4 ± 5.0 | 84.4 ± 9.4 | 81.7 ± 10.8 | <0.001 | 1.054 |
| HbA1c, % | 5.4 ± 0.6 | 5.2 ± 0.3 | 5.3 ± 0.3 | 5.4 ± 0.6 | 5.8 ± 0.9 | <0.001 | 0.553 |
| Glucose, mg/dl | 92.1 ± 21.8 | 84.0 ± 8.8 | 88.6 ± 11.0 | 92.2 ± 20.9 | 100.7 ± 35.3 | <0.001 | 0.431 |
| TC, mg/dl | 220.0 ± 43.9 | 209.0 ± 37.0 | 212.8 ± 37.6 | 225.5 ± 43.0 | 190.5 ± 46.3 | <0.001 | 0.430 |
| Triglyceride, mg/dl | 154.5 ± 91.0 | 84.6 ± 23.9 | 92.6 ± 23.7 | 164.9 ± 92.5 | 173.9 ± 100.0 | <0.001 | 0.840 |
| HDL-C, mg/dl | 56.0 ± 14.8 | 64.9 ± 14.2 | 60.0 ± 13.7 | 55.5 ± 14.5 | 49.2 ± 13.7 | <0.001 | 0.610 |
| LDL-C, mg/dl | 137.5 ± 33.4 | 125.7 ± 27.8 | 132.2 ± 28.6 | 141.9 ± 32.9 | 116.7 ± 34.3 | <0.001 | 0.435 |
| eGFR,mL/min/1.73 m² | 95.8 ± 15.0 | 106.1 ± 12.3 | 102.2 ± 13.0 | 95.6 ± 13.9 | 84.2 ± 17.8 | <0.001 | 0.815 |
| MetS, n (%) | 60,623 (18.6%) | 0 (0.0%) | 0 (0.0%) | 49,118 (19.9%) | 11,505 (37.2%) | <0.001 | 0.663 |
| Comorbidities, n (%) |  |  |  |  |  |  |  |
| Diabetes | 16,109 (4.9%) | 0 (0.0%) | 0 (0.0%) | 9,666 (3.9%) | 6,443 (20.8%) | <0.001 | 0.426 |
| Type 1 diabetes | 1,551 (0.5%) | 0 (0.0%) | 0 (0.0%) | 908 (0.4%) | 643 (2.1%) | <0.001 | 0.123 |
| Type 2 diabetes | 15,927 (4.9%) | 0 (0.0%) | 0 (0.0%) | 9,519 (3.8%) | 6,408 (20.7%) | <0.001 | 0.424 |
| Hypertension | 85,591 (26.2%) | 0 (0.0%) | 0 (0.0%) | 66,528 (26.9%) | 19,063 (61.6%) | <0.001 | 1.008 |
| Dyslipidemia | 47,757 (14.6%) | 816 (3.1%) | 922 (4.3%) | 31,270 (12.6%) | 14,749 (47.7%) | <0.001 | 0.648 |
| Cardiovascular disease | 27,679 (8.5%) | 919 (3.4%) | 0 (0.0%) | 0 (0.0%) | 26,760 (86.5%) | <0.001 | 1.790 |
| Malignancy | 29,195 (9.0%) | 2,176 (8.2%) | 1,686 (7.9%) | 21,764 (8.8%) | 3,569 (11.5%) | <0.001 | 0.065 |
| CKD | 815 (0.2%) | 3 (0.0%) | 1 (0.0%) | 250 (0.1%) | 561 (1.8%) | <0.001 | 0.108 |
| Heart failure | 1,630 (0.5%) | 39 (0.1%) | 0 (0.0%) | 0 (0.0%) | 1,591 (5.1%) | <0.001 | 0.180 |
| PAOD | 4,356 (1.3%) | 348 (1.3%) | 0 (0.0%) | 0 (0.0%) | 4,008 (13.0%) | <0.001 | 0.314 |
| Stroke | 4,956 (1.5%) | 139 (0.5%) | 0 (0.0%) | 0 (0.0%) | 4,817 (15.6%) | <0.001 | 0.333 |
| Atrial fibrillation | 5,399 (1.7%) | 166 (0.6%) | 0 (0.0%) | 0 (0.0%) | 5,233 (16.9%) | <0.001 | 0.350 |
| Hx of pancreatitis | 173 (0.1%) | 8 (0.0%) | 6 (0.0%) | 111 (0.0%) | 48 (0.2%) | <0.001 | 0.023 |
| IPMN | 34 (0.0%) | 0 (0.0%) | 0 (0.0%) | 28 (0.0%) | 6 (0.0%) | 0.056 | 0.013 |

Values are presented as mean ± standard deviation (SD) for continuous variables and number (percentage) for categorical variables. Comparisons between groups were performed using Student’s t-test for continuous variables and chi-square test for categorical variables.

Abbreviations: BMI, body mass index; SBP, systolic blood pressure; DBP, diastolic blood pressure; HbA1c, glycated hemoglobin; TC, total cholesterol; HDL-C, high-density lipoprotein cholesterol; LDL-C, low-density lipoprotein cholesterol; eGFR, estimated glomerular filtration rate; MetS, metabolic syndrome; CKD, chronic kidney disease; PAOD, peripheral arterial occlusive disease; IPMN, intraductal papillary mucinous neoplasm

**Table S4. Baseline characteristics according to pancreatic cancer development within each CKM syndrome stage**

| **Characteristics** | **CKM stage 0** | | | **CKM stage 1** | | | **CKM stage 2** | | | **CKM stage 3 or 4** | | |
| --- | --- | --- | --- | --- | --- | --- | --- | --- | --- | --- | --- | --- |
|  | **Did not develop pancreatic cancer** | **Developed pancreatic cancer** | **SMD** | **Did not develop pancreatic cancer** | **Developed pancreatic cancer** | **SMD** | **Did not develop pancreatic cancer** | **Developed pancreatic cancer** | **SMD** | **Did not develop pancreatic cancer** | **Developed pancreatic cancer** | **SMD** |
| n | 26,636 | 33 |  | 21,219 | 57 |  | 246,325 | 953 |  | 30,734 | 191 |  |
| Age, year | 51.6 ± 7.7 | 58.7 ± 7.1 | 0.967 | 52.5 ± 7.9 | 58.7 ± 7.2 | 0.824 | 56.5 ± 7.9 | 60.8 ± 6.2 | 0.606 | 61.7 ± 6.4 | 63.7 ± 4.7 | 0.350 |
| Sex, n (%) |  |  | 0.192 |  |  | 0.084 |  |  | 0.111 |  |  | 0.032 |
| Female | 20,063 (75.3%) | 22 (66.7%) |  | 14,976 (70.6%) | 38 (66.7%) |  | 122,715 (49.8%) | 422 (44.3%) |  | 10,442 (34.0%) | 62 (32.5%) |  |
| Male | 6,573 (24.7%) | 11 (33.3%) |  | 6,243 (29.4%) | 19 (33.3%) |  | 123,610 (50.2%) | 531 (55.7%) |  | 20,292 (66.0%) | 129 (67.5%) |  |
| Ethnicity, n (%) |  |  | 0.107 |  |  | 0.071 |  |  | 0.055 |  |  | 0.062 |
| Non-white | 999 (3.8%) | 2 (6.1%) |  | 1,479 (7.0%) | 3 (5.3%) |  | 12,065 (4.9%) | 36 (3.8%) |  | 1,510 (4.9%) | 7 (3.7%) |  |
| White | 25,637 (96.2%) | 31 (93.9%) |  | 19,740 (93.0%) | 54 (94.7%) |  | 234,260 (95.1%) | 917 (96.2%) |  | 29,224 (95.1%) | 184 (96.3%) |  |
| Town deprivation index | -1.5 ± 3.0 | -2.0 ± 2.0 | 0.228 | -1.3 ± 3.0 | -1.4 ± 3.1 | 0.024 | -1.5 ± 3.0 | -1.4 ± 3.2 | 0.034 | -0.9 ± 3.3 | -1.1 ± 3.3 | 0.051 |
| Household income, GBP/year |  |  | 0.390 |  |  | 0.278 |  |  | 0.237 |  |  | 0.244 |
| <18,000 | 3,124 (11.7%) | 5 (15.2%) |  | 2,960 (13.9%) | 10 (17.5%) |  | 44,381 (18.0%) | 222 (23.3%) |  | 10,016 (32.6%) | 67 (35.1%) |  |
| 18,000–30,999 | 4,798 (18.0%) | 10 (30.3%) |  | 4,113 (19.4%) | 15 (26.3%) |  | 55,170 (22.4%) | 226 (23.7%) |  | 7,442 (24.2%) | 60 (31.4%) |  |
| 31,000–51,999 | 6,661 (25.0%) | 8 (24.2%) |  | 5,490 (25.9%) | 12 (21.1%) |  | 59,263 (24.1%) | 230 (24.1%) |  | 5,138 (16.7%) | 27 (14.1%) |  |
| 52,000–100,000 | 6,918 (26.0%) | 5 (15.2%) |  | 4,995 (23.5%) | 9 (15.8%) |  | 47,386 (19.2%) | 115 (12.1%) |  | 3,017 (9.8%) | 10 (5.2%) |  |
| >100,000 | 2,526 (9.5%) | 3 (9.1%) |  | 1,598 (7.5%) | 4 (7.0%) |  | 12,194 (5.0%) | 33 (3.5%) |  | 752 (2.4%) | 5 (2.6%) |  |
| Unknown | 2,609 (9.8%) | 2 (6.1%) |  | 2,063 (9.7%) | 7 (12.3%) |  | 27,931 (11.3%) | 127 (13.3%) |  | 4,369 (14.2%) | 22 (11.5%) |  |
| Alcohol, n (%) |  |  | 0.008 |  |  | 0.274 |  |  | 0.029 |  |  | 0.045 |
| Never | 841 (3.2%) | 1 (3.0%) |  | 857 (4.0%) | 5 (8.8%) |  | 9,390 (3.8%) | 32 (3.4%) |  | 1,681 (5.5%) | 12 (6.3%) |  |
| Former | 787 (3.0%) | 1 (3.0%) |  | 656 (3.1%) | 4 (7.0%) |  | 7,798 (3.2%) | 33 (3.5%) |  | 1,893 (6.2%) | 13 (6.8%) |  |
| Current | 25,008 (93.9%) | 31 (93.9%) |  | 19,706 (92.9%) | 48 (84.2%) |  | 229,137 (93.0%) | 888 (93.2%) |  | 27,160 (88.4%) | 166 (86.9%) |  |
| Smoking, n (%) |  |  | 0.118 |  |  | 0.092 |  |  | 0.157 |  |  | 0.080 |
| Never | 16,629 (62.4%) | 21 (63.6%) |  | 12,322 (58.1%) | 34 (59.6%) |  | 136,329 (55.3%) | 467 (49.0%) |  | 12,212 (39.7%) | 71 (37.2%) |  |
| Former | 7,530 (28.3%) | 8 (24.2%) |  | 6,722 (31.7%) | 16 (28.1%) |  | 85,808 (34.8%) | 351 (36.8%) |  | 14,046 (45.7%) | 87 (45.5%) |  |
| Current | 2,477 (9.3%) | 4 (12.1%) |  | 2,175 (10.3%) | 7 (12.3%) |  | 24,188 (9.8%) | 135 (14.2%) |  | 4,476 (14.6%) | 33 (17.3%) |  |
| Physical activity, MET-min/wk | 2,725.0 ± 2,580.4 | 2,716.2 ± 2,234.3 | 0.004 | 2,650.1 ± 2,625.5 | 2,561.0 ± 2,532.2 | 0.035 | 2,666.7 ± 2,736.2 | 2,743.9 ± 2,916.5 | 0.027 | 2,508.8 ± 2,714.3 | 2,700.7 ± 2,575.5 | 0.073 |
| BMI, kg/m² | 22.4 ± 1.7 | 22.3 ± 1.5 | 0.057 | 26.9 ± 3.3 | 26.8 ± 4.3 | 0.044 | 27.6 ± 4.6 | 28.3 ± 5.0 | 0.147 | 29.1 ± 5.0 | 29.4 ± 5.2 | 0.065 |
| Waist, cm | 75.7 ± 7.5 | 76.4 ± 8.1 | 0.088 | 86.1 ± 10.1 | 86.9 ± 12.2 | 0.072 | 91.3 ± 12.9 | 94.4 ± 13.8 | 0.232 | 97.4 ± 13.7 | 98.7 ± 13.8 | 0.095 |
| SBP, mmHg | 115.8 ± 8.3 | 118.4 ± 8.4 | 0.315 | 117.7 ± 7.6 | 117.4 ± 6.8 | 0.047 | 141.0 ± 16.9 | 143.4 ± 18.0 | 0.133 | 142.5 ± 20.9 | 147.6 ± 22.1 | 0.237 |
| DBP, mmHg | 70.8 ± 5.6 | 70.0 ± 6.0 | 0.130 | 72.4 ± 5.0 | 71.3 ± 5.7 | 0.200 | 84.4 ± 9.4 | 84.4 ± 10.1 | 0.002 | 81.7 ± 10.8 | 82.9 ± 10.3 | 0.115 |
| HbA1c, % | 5.2 ± 0.3 | 5.3 ± 0.2 | 0.462 | 5.3 ± 0.3 | 5.5 ± 0.4 | 0.559 | 5.4 ± 0.6 | 5.6 ± 0.8 | 0.265 | 5.8 ± 0.9 | 6.0 ± 0.9 | 0.186 |
| Glucose, mg/dl | 84.0 ± 8.8 | 85.7 ± 8.0 | 0.210 | 88.6 ± 11.0 | 88.6 ± 10.6 | 0.001 | 92.1 ± 20.9 | 96.4 ± 28.6 | 0.170 | 100.6 ± 35.3 | 107.2 ± 39.8 | 0.176 |
| TC, mg/dl | 209.0 ± 37.0 | 210.8 ± 40.8 | 0.046 | 212.7 ± 37.6 | 217.5 ± 41.4 | 0.121 | 225.5 ± 43.0 | 223.1 ± 45.5 | 0.055 | 190.5 ± 46.3 | 188.9 ± 46.8 | 0.035 |
| Triglyceride, mg/dl | 84.5 ± 23.9 | 90.0 ± 26.4 | 0.220 | 92.6 ± 23.7 | 91.4 ± 22.8 | 0.051 | 164.9 ± 92.5 | 174.4 ± 95.8 | 0.101 | 173.9 ± 100.0 | 174.2 ± 97.6 | 0.003 |
| HDL-C, mg/dl | 64.9 ± 14.2 | 62.6 ± 15.6 | 0.151 | 60.0 ± 13.7 | 61.8 ± 16.0 | 0.122 | 55.5 ± 14.5 | 54.6 ± 14.6 | 0.066 | 49.2 ± 13.7 | 48.8 ± 13.3 | 0.032 |
| LDL-C, mg/dl | 125.7 ± 27.8 | 128.6 ± 29.4 | 0.100 | 132.2 ± 28.6 | 135.6 ± 27.4 | 0.120 | 141.9 ± 32.9 | 140.0 ± 34.6 | 0.055 | 116.7 ± 34.3 | 115.4 ± 34.2 | 0.036 |
| eGFR,mL/min/1.73 m² | 106.1 ± 12.3 | 102.1 ± 11.8 | 0.333 | 102.2 ± 13.0 | 96.4 ± 10.7 | 0.492 | 95.7 ± 13.9 | 91.0 ± 13.3 | 0.338 | 84.2 ± 17.8 | 80.6 ± 18.5 | 0.198 |
| MetS, n (%) | 0 (0.0%) | 0 (0.0%) |  | 0 (0.0%) | 0 (0.0%) |  | 48,877 (19.8%) | 241 (25.3%) | 0.131 | 11,424 (37.2%) | 81 (42.4%) | 0.107 |
| Comorbidities, n (%) |  |  |  |  |  |  |  |  |  |  |  |  |
| Diabetes | 0 (0.0%) | 0 (0.0%) |  | 0 (0.0%) | 0 (0.0%) |  | 9,593 (3.9%) | 73 (7.7%) | 0.162 | 6,385 (20.8%) | 58 (30.4%) | 0.221 |
| Type 1 diabetes | 0 (0.0%) | 0 (0.0%) |  | 0 (0.0%) | 0 (0.0%) |  | 903 (0.4%) | 5 (0.5%) | 0.024 | 638 (2.1%) | 5 (2.6%) | 0.036 |
| Type 2 diabetes | 0 (0.0%) | 0 (0.0%) |  | 0 (0.0%) | 0 (0.0%) |  | 9,447 (3.8%) | 72 (7.6%) | 0.161 | 6,350 (20.7%) | 58 (30.4%) | 0.224 |
| Hypertension | 0 (0.0%) | 0 (0.0%) |  | 0 (0.0%) | 0 (0.0%) |  | 66,200 (26.9%) | 328 (34.4%) | 0.164 | 18,926 (61.6%) | 137 (71.7%) | 0.217 |
| Dyslipidemia | 816 (3.1%) | 0 (0.0%) | 0.251 | 920 (4.3%) | 2 (3.5%) | 0.043 | 31,102 (12.6%) | 168 (17.6%) | 0.140 | 14,652 (47.7%) | 97 (50.8%) | 0.062 |
| Cardiovascular disease | 918 (3.4%) | 1 (3.0%) | 0.024 | 0 (0.0%) | 0 (0.0%) |  | 0 (0.0%) | 0 (0.0%) |  | 26,612 (86.6%) | 148 (77.5%) | 0.239 |
| Malignancy | 2,171 (8.2%) | 5 (15.2%) | 0.220 | 1,684 (7.9%) | 2 (3.5%) | 0.191 | 21,636 (8.8%) | 128 (13.4%) | 0.148 | 3,546 (11.5%) | 23 (12.0%) | 0.016 |
| CKD | 3 (0.0%) | 0 (0.0%) | 0.015 | 1 (0.0%) | 0 (0.0%) | 0.010 | 250 (0.1%) | 0 (0.0%) | 0.045 | 554 (1.8%) | 7 (3.7%) | 0.114 |
| Coronary heart disease | 323 (1.2%) | 0 (0.0%) | 0.157 | 0 (0.0%) | 0 (0.0%) |  | 0 (0.0%) | 0 (0.0%) |  | 16,491 (53.7%) | 87 (45.5%) | 0.163 |
| Heart failure | 39 (0.1%) | 0 (0.0%) | 0.054 | 0 (0.0%) | 0 (0.0%) |  | 0 (0.0%) | 0 (0.0%) |  | 1,579 (5.1%) | 12 (6.3%) | 0.049 |
| PAOD | 348 (1.3%) | 0 (0.0%) | 0.163 | 0 (0.0%) | 0 (0.0%) |  | 0 (0.0%) | 0 (0.0%) |  | 3,990 (13.0%) | 18 (9.4%) | 0.113 |
| Stroke | 139 (0.5%) | 0 (0.0%) | 0.102 | 0 (0.0%) | 0 (0.0%) |  | 0 (0.0%) | 0 (0.0%) |  | 4,786 (15.6%) | 31 (16.2%) | 0.018 |
| Atrial fibrillation | 165 (0.6%) | 1 (3.0%) | 0.181 | 0 (0.0%) | 0 (0.0%) |  | 0 (0.0%) | 0 (0.0%) |  | 5,201 (16.9%) | 32 (16.8%) | 0.005 |
| Hx of pancreatitis | 8 (0.0%) | 0 (0.0%) | 0.025 | 6 (0.0%) | 0 (0.0%) | 0.024 | 106 (0.0%) | 5 (0.5%) | 0.091 | 47 (0.2%) | 1 (0.5%) | 0.064 |
| IPMN | 0 (0.0%) | 0 (0.0%) |  | 0 (0.0%) | 0 (0.0%) |  | 27 (0.0%) | 1 (0.1%) | 0.039 | 6 (0.0%) | 0 (0.0%) | 0.020 |

Values are presented as mean ± standard deviation (SD) for continuous variables and number (percentage) for categorical variables. Comparisons between groups were performed using Student's t-test for continuous variables and chi-square test for categorical variables.

Abbreviations: BMI, body mass index; SBP, systolic blood pressure; DBP, diastolic blood pressure; HbA1c, glycated hemoglobin; TC, total cholesterol; HDL-C, high-density lipoprotein cholesterol; LDL-C, low-density lipoprotein cholesterol; eGFR, estimated glomerular filtration rate; MetS, metabolic syndrome; CKD, chronic kidney disease; PAOD, peripheral arterial occlusive disease; IPMN, intraductal papillary mucinous neoplasm

**Table S5. Baseline characteristics of study participants according to the development of pancreatic cancer (excluding baseline all cancer cases)**

| **Characteristics** | **Total** | **Did not develop  pancreatic cancer** | **Developed  pancreatic cancer** | ***P*-value** | **SMD** |
| --- | --- | --- | --- | --- | --- |
| n | 296,953 | 295,877 | 1,076 |  |  |
| Age, year | 56.02 ± 8.12 | 56.00 ± 8.12 | 60.84 ± 6.22 | <0.001 | 0.669 |
| Sex, n (%) |  |  |  | <0.001 | 0.159 |
| Female | 150,593 (50.7%) | 150,132 (50.7%) | 461 (42.8%) |  |  |
| Male | 146,360 (49.3%) | 145,745 (49.3%) | 615 (57.2%) |  |  |
| Ethnicity, n (%) |  |  |  | 0.088 | 0.058 |
| Non-white | 15,430 (5.2%) | 15,387 (5.2%) | 43 (4.0%) |  |  |
| White | 281,523 (94.8%) | 280,490 (94.8%) | 1,033 (96.0%) |  |  |
| Town deprivation index | -1.40 ± 3.03 | -1.40 ± 3.03 | -1.32 ± 3.16 | 0.400 | 0.026 |
| Household income, GBP/year |  |  |  | <0.001 | 0.270 |
| <18,000 | 54,026 (18.2%) | 53,767 (18.2%) | 259 (24.1%) |  |  |
| 18,000–30,999 | 64,631 (21.8%) | 64,359 (21.8%) | 272 (25.3%) |  |  |
| 31,000–51,999 | 70,669 (23.8%) | 70,421 (23.8%) | 248 (23.0%) |  |  |
| 52,000–100,000 | 58,233 (19.6%) | 58,109 (19.6%) | 124 (11.5%) |  |  |
| >100,000 | 16,053 (5.4%) | 16,013 (5.4%) | 40 (3.7%) |  |  |
| Unknown | 33,341 (11.2%) | 33,208 (11.2%) | 133 (12.4%) |  |  |
| Alcohol, n (%) |  |  |  | 0.754 | 0.023 |
| Never | 11,703 (3.9%) | 11,658 (3.9%) | 45 (4.2%) |  |  |
| Former | 10,033 (3.4%) | 9,993 (3.4%) | 40 (3.7%) |  |  |
| Current | 275,217 (92.7%) | 274,226 (92.7%) | 991 (92.1%) |  |  |
| Smoking, n (%) |  |  |  | <0.001 | 0.182 |
| Never | 163,293 (55.0%) | 162,786 (55.0%) | 507 (47.1%) |  |  |
| Former | 103,031 (34.7%) | 102,624 (34.7%) | 407 (37.8%) |  |  |
| Current | 30,629 (10.3%) | 30,467 (10.3%) | 162 (15.1%) |  |  |
| Physical activity, MET-min/wk | 2,662.2 ± 2,724.0 | 2,662.0 ± 2,723.6 | 2,736.4 ± 2,815.6 | 0.387 | 0.027 |
| BMI, kg/m² | 27.3 ± 4.7 | 27.3 ± 4.7 | 28.4 ± 5.1 | <0.001 | 0.217 |
| Waist, cm | 90.3 ± 13.4 | 90.3 ± 13.4 | 94.6 ± 14.3 | <0.001 | 0.307 |
| SBP, mmHg | 137.5 ± 18.5 | 137.5 ± 18.5 | 142.1 ± 19.4 | <0.001 | 0.243 |
| DBP, mmHg | 82.3 ± 10.1 | 82.3 ± 10.1 | 83.3 ± 10.5 | 0.003 | 0.093 |
| HbA1c, % | 5.4 ± 0.6 | 5.4 ± 0.6 | 5.7 ± 0.8 | <0.001 | 0.313 |
| Glucose, mg/dl | 92.0 ± 21.9 | 92.0 ± 21.8 | 97.4 ± 30.6 | <0.001 | 0.206 |
| TC, mg/dl | 219.8 ± 43.8 | 219.8 ± 43.8 | 217.0 ± 47.0 | 0.049 | 0.062 |
| Triglyceride, mg/dl | 154.4 ± 91.2 | 154.3 ± 91.2 | 167.2 ± 92.1 | <0.001 | 0.141 |
| HDL-C, mg/dl | 55.8 ± 14.7 | 55.8 ± 14.7 | 54.1 ± 14.9 | <0.001 | 0.114 |
| LDL-C, mg/dl | 137.5 ± 33.3 | 137.50± 33.3 | 135.6 ± 35.3 | 0.086 | 0.054 |
| eGFR,mL/min/1.73 m² | 96.10± 14.8 | 96.1 ± 14.8 | 90.5 ± 14.5 | <0.001 | 0.385 |
| MetS, n (%) | 55,571 (18.7%) | 55,285 (18.7%) | 286 (26.6%) | <0.001 | 0.190 |
| Comorbidities, n (%) |  |  |  |  |  |
| Diabetes | 14,551 (4.9%) | 14,434 (4.9%) | 117 (10.9%) | <0.001 | 0.224 |
| Type 1 diabetes | 1,395 (0.5%) | 1,388 (0.5%) | 7 (0.7%) | 0.519 | 0.024 |
| Type 2 diabetes | 14,383 (4.8%) | 14,267 (4.8%) | 116 (10.8%) | <0.001 | 0.224 |
| Hypertension | 76,870 (25.9%) | 76,464 (25.8%) | 406 (37.7%) | <0.001 | 0.257 |
| Dyslipidemia | 42,997 (14.5%) | 42,755 (14.5%) | 242 (22.5%) | <0.001 | 0.208 |
| Cardiovascular disease | 24,594 (8.3%) | 24,462 (8.3%) | 132 (12.3%) | <0.001 | 0.132 |
| Malignancy | 0 (0.0%) | 0 (0.0%) | 0 (0.0%) |  |  |
| CKD | 669 (0.2%) | 664 (0.2%) | 5 (0.5%) | 0.181 | 0.041 |
| Heart failure | 1,442 (0.5%) | 1,432 (0.5%) | 10 (0.9%) | 0.060 | 0.053 |
| PAOD | 3,853 (1.3%) | 3,836 (1.3%) | 17 (1.6%) | 0.493 | 0.024 |
| Stroke | 4,360 (1.5%) | 4,333 (1.5%) | 27 (2.5%) | 0.007 | 0.075 |
| Atrial fibrillation | 4,715 (1.6%) | 4,685 (1.6%) | 30 (2.8%) | 0.002 | 0.082 |
| Hx of pancreatitis | 156 (0.1%) | 152 (0.1%) | 4 (0.4%) | <0.001 | 0.070 |
| IPMN | 15 (0.0%) | 14 (0.0%) | 1 (0.1%) | 0.055 | 0.040 |
| CKM syndrome |  |  |  | <0.001 | 0.323 |
| Stage 0 | 24,493 (8.2%) | 24,465 (8.3%) | 28 (2.6%) |  |  |
| Stage 1 | 19,590 (6.6%) | 19,535 (6.6%) | 55 (5.1%) |  |  |
| Stage 2 | 225,514 (75.9%) | 224,689 (75.9%) | 825 (76.7%) |  |  |
| Stage 3 | 3,592 (1.2%) | 3,555 (1.2%) | 37 (3.4%) |  |  |
| Stage 4 | 23,764 (8.0%) | 23,633 (8.0%) | 131 (12.2%) |  |  |

Values are presented as mean ± standard deviation (SD) for continuous variables and number (percentage) for categorical variables. Comparisons between groups were performed using Student’s t-test for continuous variables and chi-square test for categorical variables.

Abbreviations: BMI, body mass index; SBP, systolic blood pressure; DBP, diastolic blood pressure; HbA1c, glycated hemoglobin; TC, total cholesterol; HDL-C, high-density lipoprotein cholesterol; LDL-C, low-density lipoprotein cholesterol; eGFR, estimated glomerular filtration rate; MetS, metabolic syndrome; CKD, chronic kidney disease; PAOD, peripheral arterial occlusive disease; IPMN, intraductal papillary mucinous neoplasm

**Table S6. Baseline characteristics of study participants according to CKM syndrome stage at baseline (excluding baseline all cancer cases)**

| **Characteristics** | **Total** | **CKM syndrome stage 0** | **CKM syndrome stage 1** | **CKM syndrome stage 2** | **CKM syndrome stage 3 or 4** | ***P*-value** | **SMD** |
| --- | --- | --- | --- | --- | --- | --- | --- |
| n | 296,953 | 24,493 | 19,590 | 225,514 | 27,356 |  |  |
| Age, year | 56.0 ± 8.1 | 51.4 ± 7.6 | 52.3 ± 7.9 | 56.2 ± 7.9 | 61.5 ± 6.4 | <0.001 | 0.783 |
| Sex, n (%) |  |  |  |  |  | <0.001 | 0.523 |
| Female | 150,593 (50.7%) | 18,297 (74.7%) | 13,666 (69.8%) | 109,586 (48.6%) | 9,044 (33.1%) |  |  |
| Male | 146,360 (49.3%) | 6,196 (25.3%) | 5,924 (30.2%) | 115,928 (51.4%) | 18,312 (66.9%) |  |  |
| Ethnicity, n (%) |  |  |  |  |  | <0.001 | 0.075 |
| Non-white | 15,430 (5.2%) | 958 (3.9%) | 1,438 (7.3%) | 11,615 (5.2%) | 1,419 (5.2%) |  |  |
| White | 281,523 (94.8%) | 23,535 (96.1%) | 18,152 (92.7%) | 213,899 (94.8%) | 25,937 (94.8%) |  |  |
| Town deprivation index | -1.4 ± 3.0 | -1.5 ± 3.0 | -1.3 ± 3.0 | -1.5 ± 3.0 | -0.9 ± 3.3 | <0.001 | 0.095 |
| Household income, GBP/year |  |  |  |  |  | <0.001 | 0.411 |
| <18,000 | 54,026 (18.2%) | 2,826 (11.5%) | 2,679 (13.7%) | 39,671 (17.6%) | 8,850 (32.4%) |  |  |
| 18,000–30,999 | 64,631 (21.8%) | 4,320 (17.6%) | 3,745 (19.1%) | 49,959 (22.2%) | 6,607 (24.2%) |  |  |
| 31,000–51,999 | 70,669 (23.8%) | 6,124 (25.0%) | 5,074 (25.9%) | 54,876 (24.3%) | 4,595 (16.8%) |  |  |
| 52,000–100,000 | 58,233 (19.6%) | 6,464 (26.4%) | 4,686 (23.9%) | 44,329 (19.7%) | 2,754 (10.1%) |  |  |
| >100,000 | 16,053 (5.4%) | 2,368 (9.7%) | 1,505 (7.7%) | 11,503 (5.1%) | 677 (2.5%) |  |  |
| Unknown | 33,341 (11.2%) | 2,391 (9.8%) | 1,901 (9.7%) | 25,176 (11.2%) | 3,873 (14.2%) |  |  |
| Alcohol, n (%) |  |  |  |  |  | <0.001 | 0.106 |
| Never | 11,703 (3.9%) | 774 (3.2%) | 805 (4.1%) | 8,632 (3.8%) | 1,492 (5.5%) |  |  |
| Former | 10,033 (3.4%) | 721 (2.9%) | 593 (3.0%) | 7,033 (3.1%) | 1,686 (6.2%) |  |  |
| Current | 275,217 (92.7%) | 22,998 (93.9%) | 18,192 (92.9%) | 209,849 (93.1%) | 24,178 (88.4%) |  |  |
| Smoking, n (%) |  |  |  |  |  | <0.001 | 0.246 |
| Never | 163,293 (55.0%) | 15,377 (62.8%) | 11,485 (58.6%) | 125,505 (55.7%) | 10,926 (39.9%) |  |  |
| Former | 103,031 (34.7%) | 6,853 (28.0%) | 6,105 (31.2%) | 77,668 (34.4%) | 12,405 (45.3%) |  |  |
| Current | 30,629 (10.3%) | 2,263 (9.2%) | 2,000 (10.2%) | 22,341 (9.9%) | 4,025 (14.7%) |  |  |
| Physical activity, MET-min/wk | 2,662.2 ± 2,724.0 | 2,720.5 ± 2,578.9 | 2,656.7 ± 2,632.9 | 2,673.5 ± 2,746.9 | 2,521.4 ± 2,720.3 | <0.001 | 0.038 |
| BMI, kg/m² | 27.3 ± 4.7 | 22.4 ± 1.7 | 27.0 ± 3.3 | 27.7 ± 4.6 | 29.1 ± 5.0 | <0.001 | 1.008 |
| Waist, cm | 90.3 ± 13.4 | 75.8 ± 7.5 | 86.2 ± 10.1 | 91.4 ± 12.9 | 97.5 ± 13.7 | <0.001 | 1.078 |
| SBP, mmHg | 137.5 ± 18.5 | 115.8 ± 8.3 | 117.7 ± 7.6 | 140.9 ± 16.8 | 142.4 ± 20.9 | <0.001 | 1.208 |
| DBP, mmHg | 82.3 ± 10.1 | 70.8 ± 5.5 | 72.4 ± 5.0 | 84.5 ± 9.4 | 81.7 ± 10.8 | <0.001 | 1.057 |
| HbA1c, % | 5.4 ± 0.6 | 5.2 ± 0.3 | 5.3 ± 0.3 | 5.4 ± 0.6 | 5.8 ± 1.0 | <0.001 | 0.553 |
| Glucose, mg/dl | 92.0 ± 21.9 | 84.0 ± 8.8 | 88.5 ± 11.0 | 92.1 ± 20.9 | 100.7 ± 35.7 | <0.001 | 0.430 |
| TC, mg/dl | 219.8 ± 43.8 | 208.6 ± 36.8 | 212.5 ± 37.7 | 225.2 ± 42.9 | 190.0 ± 46.1 | <0.001 | 0.435 |
| Triglyceride, mg/dl | 154.4 ± 91.2 | 84.3 ± 23.9 | 92.5 ± 23.8 | 165.0 ± 92.8 | 173.7 ± 99.8 | <0.001 | 0.841 |
| HDL-C, mg/dl | 55.8 ± 14.7 | 64.7 ± 14.2 | 59.9 ± 13.7 | 55.3 ± 14.4 | 49.1 ± 13.6 | <0.001 | 0.613 |
| LDL-C, mg/dl | 137.5 ± 33.3 | 125.5 ± 27.6 | 132.1 ± 28.6 | 141.8 ± 32.8 | 116.3 ± 34.2 | <0.001 | 0.441 |
| eGFR,mL/min/1.73 m² | 96.1 ± 14.8 | 106.3 ± 12.2 | 102.3 ± 12.9 | 95.8 ± 13.8 | 84.6 ± 17.5 | <0.001 | 0.816 |
| MetS, n (%) | 55,627 (18.7%) | 0 (0.0%) | 0 (0.0%) | 45,387 (20.1%) | 10,240 (37.4%) | <0.001 | 0.666 |
| Comorbidities, n (%) |  |  |  |  |  |  |  |
| Diabetes | 14,551 (4.9%) | 0 (0.0%) | 0 (0.0%) | 8,833 (3.9%) | 5,718 (20.9%) | <0.001 | 0.426 |
| Type 1 diabetes | 1,395 (0.5%) | 0 (0.0%) | 0 (0.0%) | 817 (0.4%) | 578 (2.1%) | <0.001 | 0.124 |
| Type 2 diabetes | 14,383 (4.8%) | 0 (0.0%) | 0 (0.0%) | 8,698 (3.9%) | 5,685 (20.8%) | <0.001 | 0.425 |
| Hypertension | 76,870 (25.9%) | 0 (0.0%) | 0 (0.0%) | 60,061 (26.6%) | 16,809 (61.4%) | <0.001 | 1.004 |
| Dyslipidemia | 42,997 (14.5%) | 733 (3.0%) | 837 (4.3%) | 28,279 (12.5%) | 13,148 (48.1%) | <0.001 | 0.654 |
| Cardiovascular disease | 24,594 (8.3%) | 830 (3.4%) | 0 (0.0%) | 0 (0.0%) | 23,764 (86.9%) | <0.001 | 1.814 |
| Malignancy | 0 (0.0%) | 0 (0.0%) | 0 (0.0%) | 0 (0.0%) | 0 (0.0%) |  |  |
| CKD | 669 (0.2%) | 1 (0.0%) | 1 (0.0%) | 208 (0.1%) | 459 (1.7%) | <0.001 | 0.103 |
| Heart failure | 1,442 (0.5%) | 37 (0.2%) | 0 (0.0%) | 0 (0.0%) | 1,405 (5.1%) | <0.001 | 0.180 |
| PAOD | 3,853 (1.3%) | 326 (1.3%) | 0 (0.0%) | 0 (0.0%) | 3,527 (12.9%) | <0.001 | 0.313 |
| Stroke | 4,360 (1.5%) | 119 (0.5%) | 0 (0.0%) | 0 (0.0%) | 4,241 (15.5%) | <0.001 | 0.331 |
| Atrial fibrillation | 4,715 (1.6%) | 143 (0.6%) | 0 (0.0%) | 0 (0.0%) | 4,572 (16.7%) | <0.001 | 0.347 |
| Hx of pancreatitis | 156 (0.1%) | 8 (0.0%) | 6 (0.0%) | 96 (0.0%) | 46 (0.2%) | <0.001 | 0.023 |
| IPMN | 15 (0.0%) | 0 (0.0%) | 0 (0.0%) | 13 (0.0%) | 2 (0.0%) | 0.435 | 0.008 |

Values are presented as mean ± standard deviation (SD) for continuous variables and number (percentage) for categorical variables. Comparisons between groups were performed using Student’s t-test for continuous variables and chi-square test for categorical variables.

Abbreviations: BMI, body mass index; SBP, systolic blood pressure; DBP, diastolic blood pressure; HbA1c, glycated hemoglobin; TC, total cholesterol; HDL-C, high-density lipoprotein cholesterol; LDL-C, low-density lipoprotein cholesterol; eGFR, estimated glomerular filtration rate; MetS, metabolic syndrome; CKD, chronic kidney disease; PAOD, peripheral arterial occlusive disease

**Table S7. Incidence rates and multivariable-adjusted subdistribution hazard ratios of pancreatic cancer by CKM syndrome stage at baseline (competing risk: death; baseline all cancer excluded)**

| CKM  stage | N | Events, n | Person-years (PY) | IR* | Model 1  SHR  (95% CI) | P | Model 2  SHR  (95% CI) | P | Model 3  SHR  (95% CI) | P | Model 4  SHR  (95% CI) | P |
| --- | --- | --- | --- | --- | --- | --- | --- | --- | --- | --- | --- | --- |
| 0 | 24,493 | 28 | 337,010 | 8.308 | Reference |  | Reference |  | Reference |  | Reference |  |
| 1 | 19,590 | 55 | 268,537 | 20.481 | 2.46  (1.56-3.88) | <0.001 | 2.25  (1.43-3.55) | <0.001 | 2.23  (1.41-3.52) | <0.001 | 2.22  (1.41-3.51) | <0.001 |
| 2 | 225,514 | 825 | 3,063,820 | 26.927 | 3.20  (2.20-4.67) | <0.001 | 2.08  (1.42-3.04) | <0.001 | 2.07  (1.41-3.02) | <0.001 | 2.06  (1.41-3.02) | <0.001 |
| 3–4 | 27,356 | 168 | 350,564 | 47.923 | 5.38  (3.61-8.03) | <0.001 | 2.35  (1.56-3.55) | <0.001 | 2.24  (1.48-3.38) | <0.001 | 2.19  (1.45-3.31) | <0.001 |
| P for trend |  |  |  |  |  | <0.001 |  | <0.001 |  | <0.001 |  | <0.001 |

*IR: Pancreatic cancer incidence rate per 100,000 person-years (PY). Values are presented as number of participants, number of incident pancreatic cancer cases, person-years of follow-up, and incidence rate per 100,000 person-years (PY). Subdistribution hazard ratios (SHRs) and 95% confidence intervals (CIs) were estimated using Fine–Gray subdistribution hazard models with death as a competing event.

Model 1: unadjusted

Model 2: adjusted for age and sex

Model 3: adjusted for age, sex, and smoking

Model 4: adjusted for age, sex, smoking, history of chronic pancreatitis, IPMN, Townsend Deprivation Index, and alcohol consumption

**Table S8. Association between surgical operability and outcomes in CKM Patients with pancreatic cancer**

| **CKM syndrome stage** | **Pancreatic cancer** | **Participants,  N** | **Death cases, n** | **Model 1** | | **Model 2** | | **Model 3** | | **Model 4** | |
| --- | --- | --- | --- | --- | --- | --- | --- | --- | --- | --- | --- |
|  |  |  |  | **HR (95% CI)** | ***P*-value** | **HR (95% CI)** | ***P*-value** | **HR (95% CI)** | ***P*-value** | **HR (95% CI)** | ***P*-value** |
| 1-4 | Non-op | 844 | 796 | Reference |  | Reference |  | Reference |  | Reference |  |
|  | No | 298,278 | 25340 | 0.04 (0.04-0.04) | <0.001 | 0.06 (0.05-0.06) | <0.001 | 0.06 (0.06-0.07) | <0.001 | 0.06 (0.06-0.07) | <0.001 |
|  | Op | 357 | 185 | 0.33 (0.28-0.39) | <0.001 | 0.36 (0.30-0.42) | <0.001 | 0.39 (0.33-0.45) | <0.001 | 0.38 (0.33-0.45) | <0.001 |
| 1 | Non-op | 34 | 31 | Reference |  | Reference |  | Reference |  | Reference |  |
|  | No | 21,219 | 813 | 0.02 (0.01-0.03) | <0.001 | 0.03 (0.02-0.05) | <0.001 | 0.03 (0.02-0.05) | <0.001 | 0.03 (0.02-0.05) | <0.001 |
|  | Op | 23 | 11 | 0.33 (0.16-0.65) | 0.001 | 0.31 (0.16-0.63) | <0.001 | 0.28 (0.14-0.57) | <0.001 | 0.27 (0.13-0.53) | <0.001 |
| 2 | Non-op | 661 | 623 | Reference |  | Reference |  | Reference |  | Reference |  |
|  | No | 246,325 | 17693 | 0.04 (0.03-0.04) | <0.001 | 0.05 (0.04-0.05) | <0.001 | 0.05 (0.05-0.06) | <0.001 | 0.05 (0.05-0.06) | <0.001 |
|  | Op | 292 | 153 | 0.34 (0.29-0.41) | <0.001 | 0.36 (0.30-0.43) | <0.001 | 0.39 (0.32-0.46) | <0.001 | 0.38 (0.32-0.46) | <0.001 |
| 3–4 | Non-op | 149 | 142 | Reference |  | Reference |  | Reference |  | Reference |  |
|  | No | 30,734 | 6834 | 0.10 (0.09-0.12) | <0.001 | 0.11 (0.10-0.13) | <0.001 | 0.12 (0.10-0.14) | <0.001 | 0.12 (0.10-0.14) | <0.001 |
|  | Op | 42 | 21 | 0.27 (0.17-0.43) | <0.001 | 0.31 (0.20-0.49) | <0.001 | 0.33 (0.21-0.52) | <0.001 | 0.34 (0.22-0.55) | <0.001 |

No, absence of pancreatic cancer; Non-op, pancreatic cancer diagnosed without corresponding operative procedure codes; Op, pancreatic cancer diagnosed with corresponding operative procedure codes. Hazard ratios (HRs) and 95% confidence intervals (CIs) for all-cause mortality were estimated using a Cox proportional hazards model, with the Non-op group as the reference category.

Model 1: unadjusted

Model 2: adjusted for age and sex

Model 3: adjusted for age, sex, and smoking

Model 4: adjusted for age, sex, smoking, history of chronic pancreatitis, IPMN, Townsend Deprivation Index, and alcohol consumption

**Table S9. Sensitivity analyses of the association between CKM syndrome stage and pancreatic cancer risk after exclusion of pancreatic cancer cases occurring within 2 years after diabetes onset**

| CKM  stage | N | Events, n | Person-years (PY) | IR* | Model 1  SHR  (95% CI) | P | Model 2  SHR  (95% CI) | P | Model 3  SHR  (95% CI) | P | Model 4  SHR  (95% CI) | P |
| --- | --- | --- | --- | --- | --- | --- | --- | --- | --- | --- | --- | --- |
| 0 | 26,666 | 33 | 366,167 | 9.012 | Reference |  | Reference |  | Reference |  | Reference |  |
| 1 | 21,259 | 55 | 290,700 | 18.920 | 2.09  (1.36-3.22) | <0.001 | 1.92  (1.24-2.95) | 0.003 | 1.90  (1.24-2.93) | 0.003 | 1.90  (1.23-2.93) | 0.004 |
| 2 | 246,591 | 888 | 3,339,495 | 26.591 | 2.91  (2.06-4.12) | <0.001 | 1.89  (1.33-2.69) | <0.001 | 1.89  (1.33-2.68) | <0.001 | 1.88  (1.32-2.67) | <0.001 |
| 3–4 | 30,667 | 178 | 390,247 | 45.612 | 4.70  (3.24-6.81) | <0.001 | 2.07  (1.41-3.04) | <0.001 | 1.99  (1.35-2.92) | <0.001 | 1.94  (1.32-2.85) | <0.001 |
| P for trend |  |  |  |  |  | <0.001 |  | <0.001 |  | <0.001 |  | <0.001 |

*IR: Pancreatic cancer incidence rate per 100,000 person-years (PY). Values are presented as number of participants, number of incident pancreatic cancer cases, person-years of follow-up, and incidence rate per 100,000 person-years (PY). Subdistribution hazard ratios (SHRs) and 95% confidence intervals (CIs) were estimated using Fine–Gray subdistribution hazard models with death as a competing event.

Model 1: unadjusted

Model 2: adjusted for age and sex

Model 3: adjusted for age, sex, and smoking

Model 4: adjusted for age, sex, smoking, history of chronic pancreatitis, IPMN, Townsend Deprivation Index, and alcohol consumption

**Table S10. Additional subgroup and sensitivity analyses evaluating the influence of baseline diabetes status on the association between CKM syndrome stage and pancreatic cancer risk**

| CKM  stage | N | Events, n | Person-years (PY) | IR* | Model 1  SHR  (95% CI) | P | Model 2  SHR  (95% CI) | P | Model 3  SHR  (95% CI) | P | Model 4  SHR  (95% CI) | P |
| --- | --- | --- | --- | --- | --- | --- | --- | --- | --- | --- | --- | --- |
| Participants without baseline diabetes | | | | |  |  |  |  |  |  |  |  |
| 0 | 26,669 | 33 | 366,206 | 9.011 | Reference |  | Reference |  | Reference |  | Reference |  |
| 1 | 21,276 | 57 | 290,931 | 19.592 | 2.17  (1.41-3.33) | <0.001 | 1.99  (1.29-3.05) | 0.002 | 1.97  (1.28-3.03) | 0.002 | 1.97  (1.28-3.02) | 0.002 |
| 2 | 246,370 | 948 | 3,335,625 | 28.420 | 3.11  (2.20-4.40) | <0.001 | 2.02  (1.42-2.88) | <0.001 | 2.01  (1.42-2.86) | <0.001 | 2.01  (1.41-2.86) | <0.001 |
| 3–4 | 30,282 | 186 | 385,887 | 48.201 | 4.98  (3.44-7.20) | <0.001 | 2.20  (1.50-3.22) | <0.001 | 2.10  (1.44-3.09) | <0.001 | 2.06  (1.40-3.02) | <0.001 |
| P for trend |  |  |  |  |  | <0.001 |  | <0.001 |  | <0.001 |  | <0.001 |
| Participants with baseline Type 2 diabetes | | | |  |  |  |  |  |  |  |  |  |
| 2 | 9,519 | 72 | 125,385 | 57.423 | Reference |  | Reference |  | Reference |  | Reference |  |
| 3–4 | 6,408 | 58 | 77,597 | 74.746 | 0.83  (0.59-1.18) | 0.307 | 1.03  (0.72-1.48) | 0.874 | 1.11  (0.76-1.62) | 0.585 | 1.10  (0.75-1.62) | 0.631 |
| P for trend |  |  |  |  |  | 0.307 |  | 0.874 |  | 0.585 |  | 0.631 |

*IR: Pancreatic cancer incidence rate per 100,000 person-years (PY). Values are presented as number of participants, number of incident pancreatic cancer cases, person-years of follow-up, and incidence rate per 100,000 person-years (PY). Subdistribution hazard ratios (SHRs) and 95% confidence intervals (CIs) were estimated using Fine–Gray subdistribution hazard models with death as a competing event.

Model 1: unadjusted

Model 2: adjusted for age and sex

Model 3: adjusted for age, sex, and smoking

Model 4: adjusted for age, sex, smoking, history of chronic pancreatitis, IPMN, Townsend Deprivation Index, and alcohol consumption

**Supplementary Figure 1. Conceptual Framework Linking CKM Severity and Pancreatic Cancer Development**

This schematic summarizes the proposed biological pathways connecting cardiovascular–kidney–metabolic syndrome progression with pancreatic cancer development, highlighting inflammatory cytokines, oxidative stress, and metabolic dysregulation
